# Shifting epigenetic contexts influence regulatory variation and disease risk

**DOI:** 10.18632/aging.203194

**Published:** 2021-06-16

**Authors:** Daniel Richard, Terence D. Capellini

**Affiliations:** 1 Department of Human Evolutionary Biology, Harvard University, Cambridge, MA 02138, USA; 2 Broad Institute of MIT and Harvard, Cambridge, MA 02142, USA

**Keywords:** development, aging, evolution, GWAS, disease

## Abstract

Epigenetic shifts are a hallmark of aging that impact transcriptional networks at regulatory level. These shifts may modify the effects of genetic regulatory variants during aging and contribute to disease pathomechanism. However, these shifts occur on the backdrop of epigenetic changes experienced throughout an individual’s development into adulthood; thus, the phenotypic, and ultimately fitness, effects of regulatory variants subject to developmental- versus aging-related epigenetic shifts may differ considerably. Natural selection therefore may act differently on variants depending on their changing epigenetic context, which we propose as a novel lens through which to consider regulatory sequence evolution and phenotypic effects. Here, we define genomic regions subjected to altered chromatin accessibility as tissues transition from their fetal to adult forms, and subsequently from early to late adulthood. Based on these epigenomic datasets, we examine patterns of evolutionary constraint and potential functional impacts of sequence variation (e.g., genetic disease risk associations). We find that while the signals observed with developmental epigenetic changes are consistent with stronger fitness consequences (i.e., negative selection pressures), they tend to have weaker effects on genetic risk associations for aging-related diseases. Conversely, we see stronger effects of variants with increased local accessibility in adult tissues, strongest in young adult when compared to old. We propose a model for how epigenetic status of a region may influence the effects of evolutionary relevant sequence variation, and suggest that such a perspective on gene regulatory networks may elucidate our understanding of aging biology.

## INTRODUCTION

It has been suggested that the process of aging, and the concomitant manifestation of aging-related disease, is subject to both genetic and non-genetic factors impacting the regulatory networks (and subsequent behaviors) of aging cells [1, 2]. Nongenetic regulation of aging refers to epigenetics; chemical changes to the genome (e.g., at the chromatin level) that impact transcriptional programs [1], and which have been shown to accumulate with age [3–5]. The epigenetic state of chromatin can be broadly classified into activating or repressing modifications [6], referring, in part, to the increased/decreased accessibility of DNA to gene-regulatory machinery (e.g., transcription factors), and is established, maintained, and reset to switch between states [6]. Ample evidence suggests a causal relationship between changes in epigenetic state with age and hallmarks of aging in cells [3, 5]. Much recent work has focused on elucidating this relationship and how, ultimately, this contributes to age-related tissue decline and adult diseases [7].

As aging can also be considered a continuation of development [8], the epigenetic changes that are retained from early-life development may have important consequences for the adult epigenome – establishing the context within which epigenetic aging occurs [9]. A ‘fetal programming’ model has been suggested whereby early epigenetic plasticity in response to environmental and nutritional stimuli, while being adaptive and beneficial to fetal and early post-natal growth, has deleterious consequences later in life by contributing to adult disease risk [9–12]. This serves as one possible mechanism for the theorized consequences of selection favoring early-life development at the cost of late-life function [9, 13, 14]. Evidence supporting this model has been largely limited to DNA methylation [9, 15–17], though replication of important loci findings has been difficult [18].

Epigenetic marks established during development can persist into adulthood [9], but they do so in the context of shifts in epigenetic states (see below) as tissues transition into their adult forms and functions. This transition process has been characterized with respect to DNA methylation, chromatin state, and gene expression across multiple tissues [19–21]. Furthermore, these fetal to adult epigenetic shifts can be compounded by additional modifications through aging-associated epigenetic changes. Such epigenetically-regulated biological pathways involved in development, such as Wnt signalling, subsequently take on a role in tissue homeostasis in adults and are implicated in age-related tissue decline [22, 23] – suggesting a molecular link between processes mediating growth and aging [8, 24]. Thus, an important component of understanding the contributions of fetal programming as well as epigenetic aging to disease biology and risk is characterizing the epigenetic changes between fetal and adult tissues and how these might interact with subsequent aging-associated modifications.

While epigenomes vary between cell types [25, 26] and changes to epigenetic state with age may be expected to manifest differently, similar aging epigenetic have been repeatedly observed across tissues epigenetic shifts have been repeatedly observed across tissues [1, 5, 20, 27, 28]. Similarly, while age-related expression changes do exhibit tissue-specificity, there is evidence of potential synchronized changes across different sets of tissues [29], particularly for certain sets of genes and pathways [29, 30], and these changes may integrate at multiple different epigenetic levels [31]. Together, these findings suggest that a central trajectory for epigenetic state that reflects innate aging processes may exist [20], upon which extrinsic and cell-type effects are layered. Similarly, studies between fetal and adult tissues have found that, while epigenetic change is observed within individual tissues, there are also common trends of development (e.g., chromatin restriction, particularly at loci involved in early development) [21, 32, 33].

Importantly, the epigenetic state of genetic variants (e.g., single nucleotide polymorphisms) influences their regulatory effects, and subsequent association with heritable disease risk [34]. Thus, general epigenetic trends across early development and later aging may influence the phenotypic effects of regulatory mutations, albeit the extent to which this occurs is unknown. These phenotypes, if impacting an individual’s fitness, may be acted upon by natural selection. Evolutionary theories have been proposed which suggest that mutations contributing to aging pathologies are ‘allowed’ to accumulate due to the reduced fitness consequences of disease in older, post-reproductive individuals [35], or that beneficial mutations selected for early development become deleterious with age [36–38]. Studying the added dimension of epigenetic context may provide a fresh perspective on theories of aging and selection. For example, deleterious mutations that change epigenetic context later in life may have different regulatory effects, and thus different fitness consequences, which alter the selective pressures acting on them.

In the present study, we seek to characterize common epigenetic trends between fetal and adult tissues, and subsequently examine the potential interaction of these developmental changes with later changes associated with epigenetic aging in adult tissues. We utilize our findings to propose a model for how evolutionary forces may have acted at these loci in humans, and how these forces in turn influence the distribution of mutations conferring heritable disease risk across a number of age-associated pathologies.

## RESULTS

### Defining chromatin accessibility change, its genomic context, and loci subject to change

To investigate epigenetic changes occurring over the course of post-natal development and aging, we focused on chromatin accessibility, as it reflects the regulatory potential of a genetic locus and can be considered a property of the epigenome which integrates a number of possible epigenetic phenomena (e.g., regulatory factor binding, chromatin remodelling, etc.) [39]. We thus consider regions with altered chromatin accessibility as being indicative of epigenetic modifications or ‘shifts’ in context. As a read-out of accessibility we analyzed DNase-I hypersensitivity datasets acquired from primary human tissue, and obtained fetal/adult sample pairs for eight distinct primary tissues (spleen, lung, muscle, stomach, kidney, brain, heart, and skin; see Supplementary Table 1) [6, 40]. For each tissue at each time-point, called accessible or “open” chromatin regions were consolidated across biological replicates, then further aggregated by tissue and stage (see Supplementary Methods).

**Figure 1:**
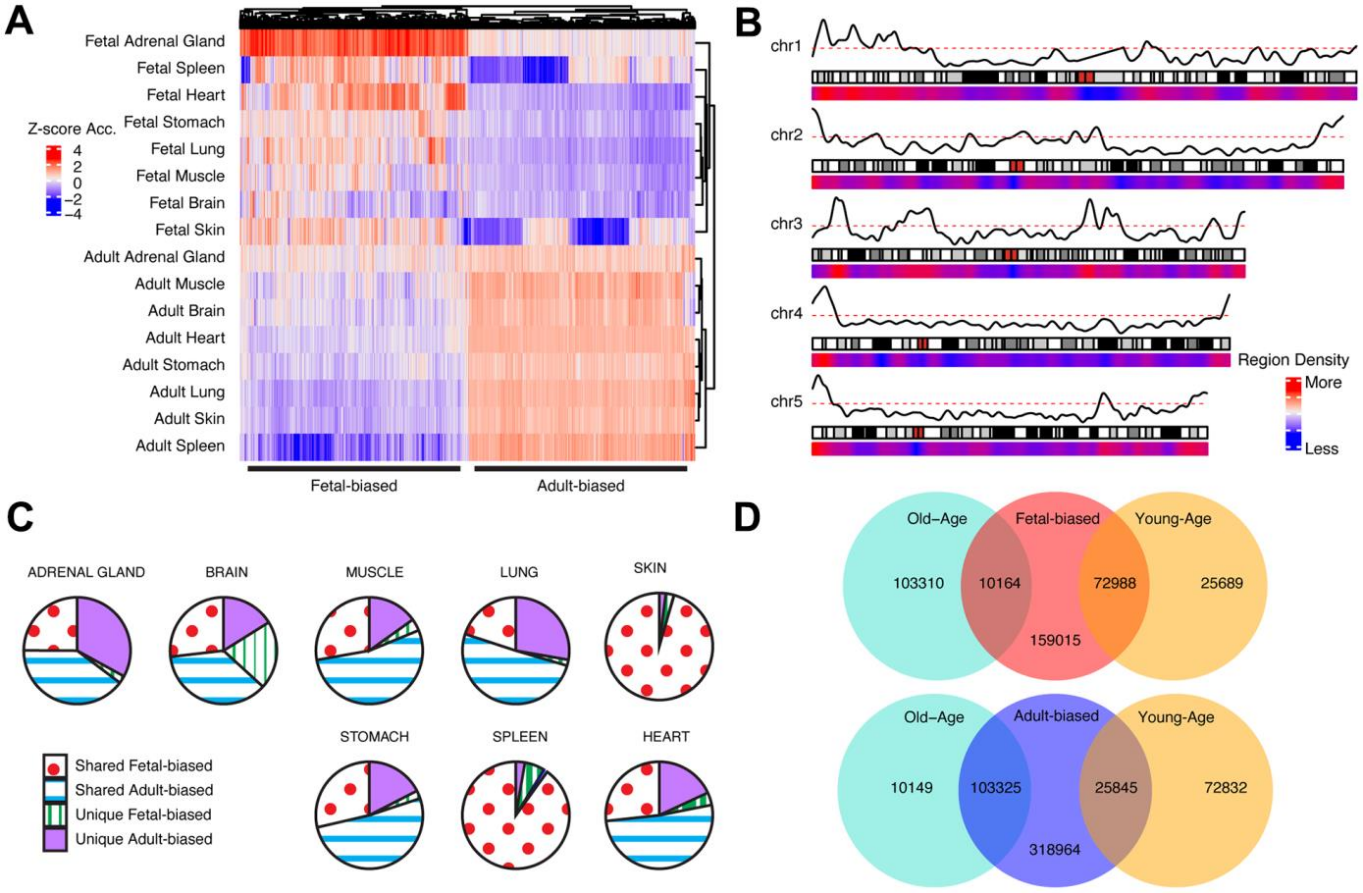
**Cross-tissue accessibility.** (**A**) Representative heatmap of Dnase-I accessibility for regions significantly different between fetal/adult tissues. Color scale indicates magnitude of chromatin accessibility signal (see Supplementary Methods). Horizontal lines denote defined fetal-biased (left) and adult-biased regions. (**B**) Genomic distribution of regions changing accessibility in fetal and adult comparison. Red/blue: density of defined differentially-accessible regions. Solid black line: relative proportion of regions more accessible in adult (top) or fetal (bottom) tissues. First five autosomes shown (see Supplementary Figure 2). (**C**) The proportion of defined altered-accessibility regions between adult and fetal samples for indicated tissues which are unique to that tissue, or captured in the pan-tissue set. (**D**) Overlaps between regions defined as differentially-accessible in fetal/adult comparison and those defined in the young/old-age comparison. Directionality in accessibility change is significantly shared (see Supplementary Table 1). Related content can be found in Supplementary Information, Supplementary Figures 1–6 and Supplementary Tables 1, 2.

We first identified chromatin regions exhibiting recurrent accessibility changes between fetal and adult samples across tissue types (see Supplementary Methods, Figure 1A, 1B and Supplementary Figures 1, 2). We define regions as ‘adult-biased’ if they exhibit increased differential accessibility in adults compared to in fetuses. Conversely, we define regions as ‘fetal-biased’ if they exhibited decreased differential accessibility in adults compared to in fetuses. These ‘pan-tissue’ altered regions were compared to those defined in individual tissue comparisons, showing substantial but not complete overlap (Figure 1C) – suggesting that our approach captures cross-tissue signals of broader developmental changes and not tissue-specific effects. We next explored possible signals of epigenetic aging occurring in the context of fetal to adult changes, by further dividing our adult tissue samples into ‘younger’ and ‘older’ age categories (Methods, Supplementary Figure 3). We then assessed accessibility change between young and old occurring within the ‘adult-biased’ and ‘fetal-biased’ regions defined above (Figure 1D). This approach identified regions for which young-old differences mirrored fetal-adult differences, as well as regions where aging changes appear to counter developmental patterns. We observed a tendency for shared directionality in gains or losses of accessibility; i.e., adult-biased regions tended to also have increased accessibility in older adult samples, while regions losing accessibility in adult samples (i.e., are fetal-biased) continued this trend in older samples (chi-sq test, p < 0.05, Supplementary Table 1). In this text, we therefore refer to regions with greater accessibility in older samples as ‘old-biased’ and regions with lower accessibility in older samples as ‘young-biased’. As described in the Supplementary Information, we considered histone mark and DNA methylation changes, key features of developmental [21, 26, 33] and aging epigenetic changes [3, 20] as additional means to validate the behavior of these region sets (see also Supplementary Table 1).

To gain insights into the roles these region sets have in transcriptional regulation, we next characterized the genomic distribution of our adult-biased and fetal-biased region sets using adult tissue epigenetic states [26] (Supplementary Methods). We found that our region sets preferentially fell within different epigenetic states (e.g., enhancers, heterochromatin) depending on the nature of their accessibility shift (e.g., adult-biased, old-biased), suggesting that these shifts may be associated with altered regulatory biology at different loci, and that the interaction between fetal and adult shifts as well as young and old-age shifts heavily favors developmental changes to accessibility (see Supplementary Figures 4, 5, Supplementary Information).

As accessible chromatin regions often serve to regulate gene expression [39] by acting as *cis*-regulatory sequences, we next sought to identify the potential role of our regions in regulatory changes occurring during development and aging. We did this by considering promoter-level accessibility (see Methods, Supplementary Figure 6), promoter-capture (Hi-C) interactions [41], and regulatory-domain annotations [42] for genes which may be subject to control by these regulatory regions. We found a general pattern for enrichment of immune-related gene sets with the adult-biased set, while development-related (e.g., cellular proliferation) terms were enriched with fetal-biased regions, patterns echoed when considering old-biased and young-biased region sets, respectively (see Supplementary Information, Supplementary Table 2).

We next incorporated tissue expression datasets looking for general gene expression trends between fetal and adult tissues (see Methods). We observe similar enrichment terms as well as significant overlaps with gene sets defined on the regulatory level (see Supplementary Information, Supplementary Table 2). Similarly, we utilized GTEx (gene tissue expression) datasets [43] to look for corresponding shifts in gene expression with age, similar to previous work [44] (see Methods)(Supplementary Table 2). While we did not observe significant overlaps between these aging-expression gene sets and those defined using aging-accessibility changes, we did see significant overlaps with the fetal/adult expression comparisons, along with enriched gene sets with relevance to aging biology (see Supplementary Information, Supplementary Table 2).

As development and aging are phenomena subjected to the actions of random and directed evolutionary forces [13, 45, 46], we next develop expectations for how these epigenetically-altered regions may have evolved over time.

### Sequence evolution of epigenetically-altered regions

**Figure 2:**
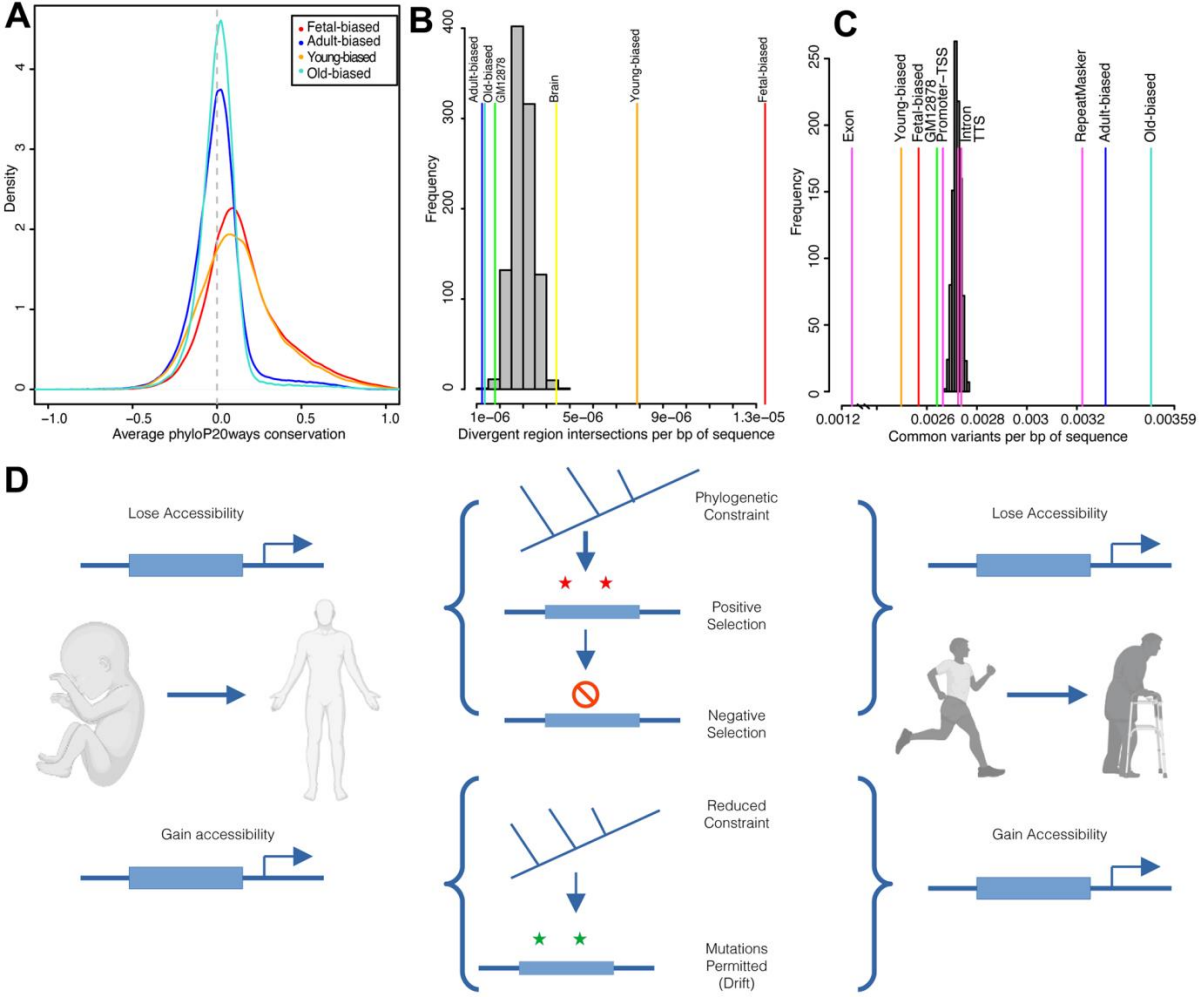
**Sequence evolution of age-altered regions.** (**A**) Distribution of average per-region sequence conservation (phyloP20ways) in differentially-accessible regions (see color legend). (**B**) Overlaps of developmental and age-related region sets and human acceleration regions. Overlaps shown relative to set size (per bp of sequence) for background (gray) and target (colored) sets; labels correspond to Supplementary Table 3. (**C**) Intersections of common human variants (per bp of sequence) for target (colored) and randomized (grey) region sets; labels correspond to results in Supplementary Table 3. (**D**) Diagram summarizing results of evolutionary sequence analyses. Accessible regions, here diagrammed as an upstream enhancer element (thick blue box), which either gain or lose accessibility over development (left) or ageing (right) exhibit different patterns of evolutionary sequence behavior. Created with BioRender.com. See also Supplementary Figures 7, 8.

Development and aging are simultaneously very ancient and variable [45, 47] biological processes and are particularly divergent in key species [48]. Thus, it may be the case that both development and age-associated regions have been shaped by a mix of evolutionary forces acting to either maintain or modify genetic sequences (e.g., regulatory enhancers). To address this possibility, we examined patterns of sequence conservation in our epigenetic datasets using phyloP [49], a measure of nucleotide conservation and/or acceleration across species (Figure 2A). Across primates, we observed that fetal-biased regions tended to have greater sequence conservation than adult-biased regions, and furthermore that both sets differed significantly from those regions not defined as developmentally-altered (Supplementary Table 3). These patterns were similarly observed when comparing age-associated regions (Supplementary Table 3). These findings of conservation differences between sets suggests that the greater regulatory and developmental role associated with fetal-biased and young-biased regions (e.g., enriched for enhancer elements) exerts functional sequence constraint while adult-biased and old-biased regions (e.g., enriched for repressed segments) are less conserved across species.

Within this broader context of species diversity and evolution, humans and chimpanzees display marked and obvious differences in development and longevity [45, 50]. This relatively recent divergence is thought to be driven largely by non-coding changes to cis-regulation [51]. We therefore next looked for evidence of regulatory modifications to biological processes that may contribute to these human/chimp differences. To do this, we intersected our regions sets with sequences demonstrating significant divergence along the human lineage (e.g., ‘human accelerated regions’ [52]). We found that fetal-biased regions were enriched for signals of acceleration while adult-biased regions were depleted (Figure 2B). Similar patterns were seen for young versus old-biased regions (Figure 2B and Supplementary Table 3). We found a number of genes involved in development and aging processes with putative nearby regulatory elements intersecting accelerated regions, two examples of which are shown in Supplementary Figure 7 (see also Supplementary Information).

To gauge the evolutionary interaction between sequence constraint across species and within-species variation, we next assessed modern-day human diversity within region sets (Methods). We found that genetic diversity in fetal-biased regions was markedly reduced (i.e., constrained) compared to genomic backgrounds, as well as to intronic and promoter-TSS elements (Figure 2C and Supplementary Table 3). Conversely, adult-biased regions were enriched for sequence diversity, at the level of annotated repeat elements. These patterns were accentuated when examining young- and old-biased region datasets, and comparing region sets directly (Supplementary Table 3, Supplementary Information). Importantly, when we considered sequence diversity within other ape species, we also observed a decrease in fetal-biased, and young-biased sequence diversity (relative to adult-biased and old-biased, respectively). This latter finding further suggests that fetal-biased regions are associated with conserved regulatory function that discourages mutation and/or drift (Supplementary Figure 8 and Supplementary Table 3).

Overall, natural selection appears to have acted upon regions subject to accessibility shifts in development and aging, modifying some loci (i.e., accelerated divergence indicative of ancient positive selection) while protecting others (i.e., reduced variation indicative of more recent negative selection) (Figure 2D).

Importantly, selective forces, both positive and negative, manifest phenotypically through the effects of random genetic mutations, which act to modify gene regulatory networks to varying degrees. We next examine this relationship.

### Epigenetic shifts in age-associated trait associations

In our above analyses, the observed signals of consistent inter- and intra-species conservation in regions most associated with early development (i.e., the fetal-biased set) follows with the expectation that variants negatively impacting early-life would be subject to stronger purifying selection [9, 53, 54]. Conversely, variants with later-manifesting effects, i.e., those within regions increasing in local accessibility with age (i.e., the adult-biased set), would be subjected to substantially weaker selection and may therefore be ‘tolerated’ [55, 56]. To test expectations of the possible deleterious effects of variants subject to accessibility change over development and aging, we utilized GWAS datasets available from the UK Biobank [57]. We extracted summary-statistics for a collection of 127 complex diseases/pathologies falling into aging-related categories [58], including metabolic disorders, cancers, cardiovascular disease, and musculoskeletal impairment (Supplementary Table 4, Methods). We similarly analyzed a set of developmental trait GWAS to act as a control for our fetal/adult accessibility comparisons, and finally considered longevity GWAS data (Supplementary Table 4, see Supplementary Information).

**Figure 3:**
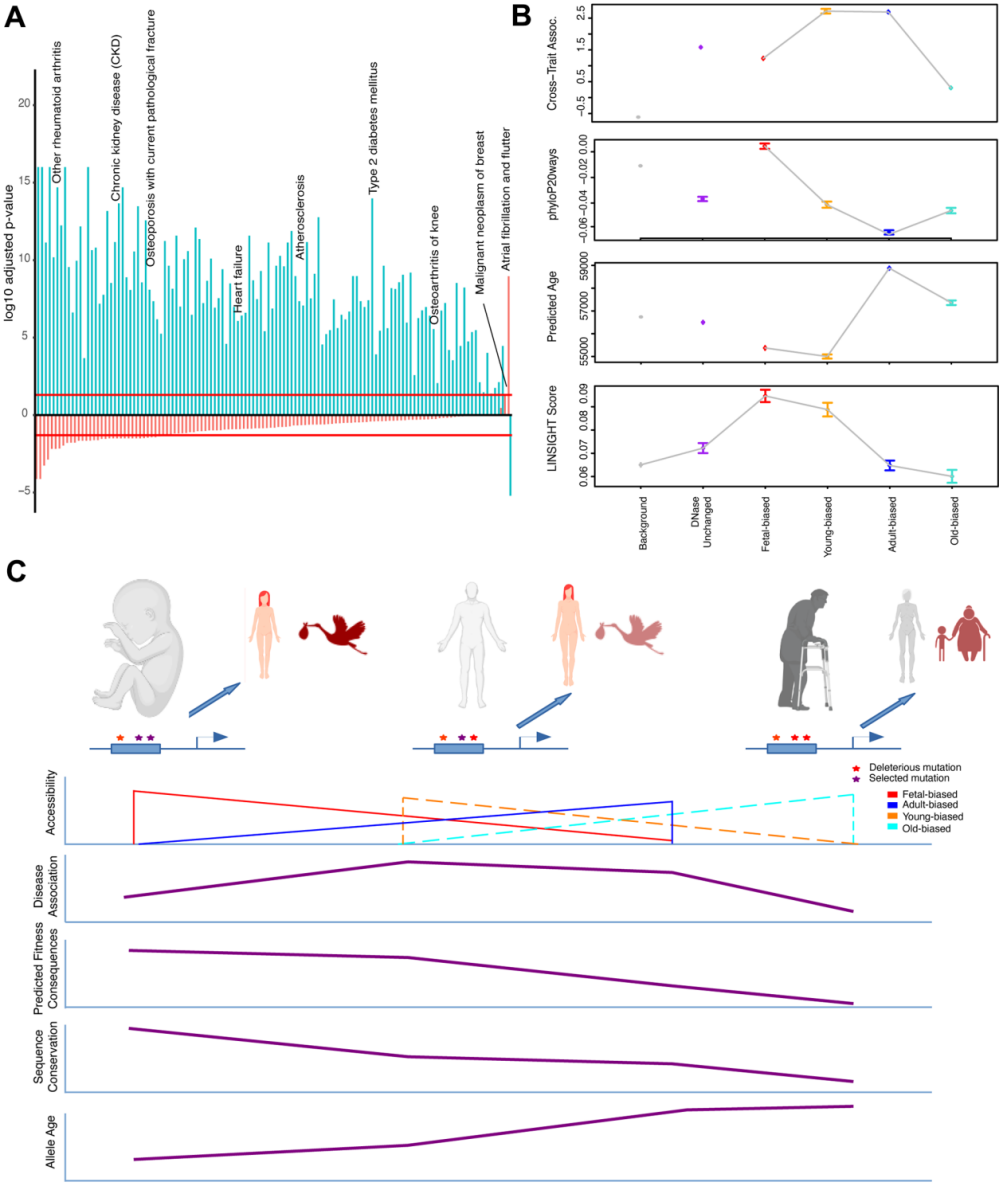
**Epigenetic context and heritable disease associations.** (**A**) Adjusted p-values for hypergeometric tests showing enrichment/depletion (positive/negative) for GWAS variants nearby regions increasing (blue) or decreasing (red) accessibility across adult tissues for a number of age-associated diseases (see Supplementary Table 4). (**B**) Cross-set disease associations, and additional per-SNP metrics, for variants nearby developmental and age-altered region sets along with unaltered DNase sites and variants not nearby accessible regions. See Supplementary Table 4 and Methods. (**C**) Model for the effects of epigenetic context on disease association and sequence evolution. (Top): Example enhancer elements more accessible in fetal, adult, and old-adult tissues (left-right) which have been modified by mutations. (Left): Deleterious mutations disrupting regulation in development stand to have the biggest impact on fitness, while having a moderate effect on tissue homeostasis. (Middle): Mutations disrupting regulation in young-adult tissues have a moderate impact on fitness, but a larger effect on tissue homeostasis (particularly over adulthood). (Right): Mutations disrupting regulation in old-adult tissues have weak impacts on fitness, and a weak effect on tissue homeostasis (which has already deteriorated with age). (Bottom): Illustrating patterns of accessibility, disease association, sequence constraint and variant allele age for these sets of regions changing accessibility over time.

It has been suggested that the highly polygenic nature of complex traits and diseases reflects cumulative regulatory modification to a ‘core’ set of genes which functions most proximately in relevant biology [59]. Across age-associated diseases, this may reflect general aging processes, and regulatory variants impacting these would be expected to contribute to heritable aging-disease risk broadly. Given this rationale, we first considered the behavior of individual SNPs nearby accessibility-altered regions across diseases, and subsequently these behaviors at the gene-locus level (below). We aggregated per-SNP associations across diseases as a singular cross-set metric of risk association (Supplementary Methods). We confirmed that ClinVar variants, variants for which possible clinical significance have been described [60], tended to be more risk-associated by this metric, as we would expect (Supplementary Table 4). Additionally, across all diseases we individually performed enrichment tests for strongly-associated variants nearby our region sets, which corroborated the cross-disease results described below (Figure 3A, see Supplementary Information).

As variants in accessible non-coding regions likely have regulatory impacts generally [39], we confirmed that variants within or nearby regions not classified as strictly developmental nor age-altered tended to have greater association than non-accessible variants. However, this control set had significantly less association than variants nearby sets of both developmentally- and age-altered regions (i.e., fetal/adult-biased, and young/old-biased regions) (Supplementary Table 4).

Considering first developmental change, we found that variants in regions gaining nearby accessibility in adults (i.e., adult-biased) have greater association with disease than those in regions losing nearby accessibility (i.e., fetal-biased) (Figure 3B). Unexpectedly, when looking at aging accessibility changes, we observed that variants in regions gaining nearby accessibility in older-samples actually have lower cross-disease associations than those in regions becoming more accessible in younger samples (Figure 3B and Supplementary Table 4). Furthermore, we found that for intersections of development and age-altered regions that the increased disease association with adult-biased regions was abrogated when intersected with old-biased regions. The magnitude of region-set differences in disease associations was also greater in the young/old-biased comparisons (see Figure 3B, Supplementary Information).

Taken together, these results would suggest that those variants most accessible in younger adults stand to have the greatest impact (in terms of association p-value) on late-life disease risk – a finding that may have important implications for understanding the development of disease over adulthood (see Figure 3C, Discussion).

We next considered these disease-association patterns at the gene-locus level. Briefly, for a given disease we assign the most significant nearby SNP to all genes, and subsequently rank genes based on their assigned GWAS signal. Gene ranks are then aggregated across diseases, looking for genes consistently ranked higher across sets (Supplementary Methods). To confirm the behavior of this gene-ranking method, we compared the cross-set ranking of genes associated with homeostatic processes (based on GO annotations) to randomized gene sets, finding that these gene loci tend to harbor stronger genetic variants across a larger number of diseases than expected (compared to randomized sets), but not so for genes involved in reproductive organ development (see Supplementary Information, Supplementary Table 4).

We applied this method to the sets of development and age-associated genes we defined above and asked whether they tended to have more or less cross-disease GWAS signals than expected. Our sets defined by accessibility-region contacts supported our earlier findings on strong GWAS signals nearby development and age-altered regions – namely, loci of both adult-biased and young-biased gene sets were enriched for strong GWAS signals across diseases, while fetal-biased and old-biased gene sets were associated with relatively weaker GWAS signals (Supplementary Table 4). Sets defined by RNA-seq data showed more of a mix of enriched/depleted GWAS signals across developmental and age comparisons, reflecting the possibility that a mixture of genes increasing and decreasing expression over time may additively contribute to aging disease biology (see Supplementary Information).

**Figure 4:**
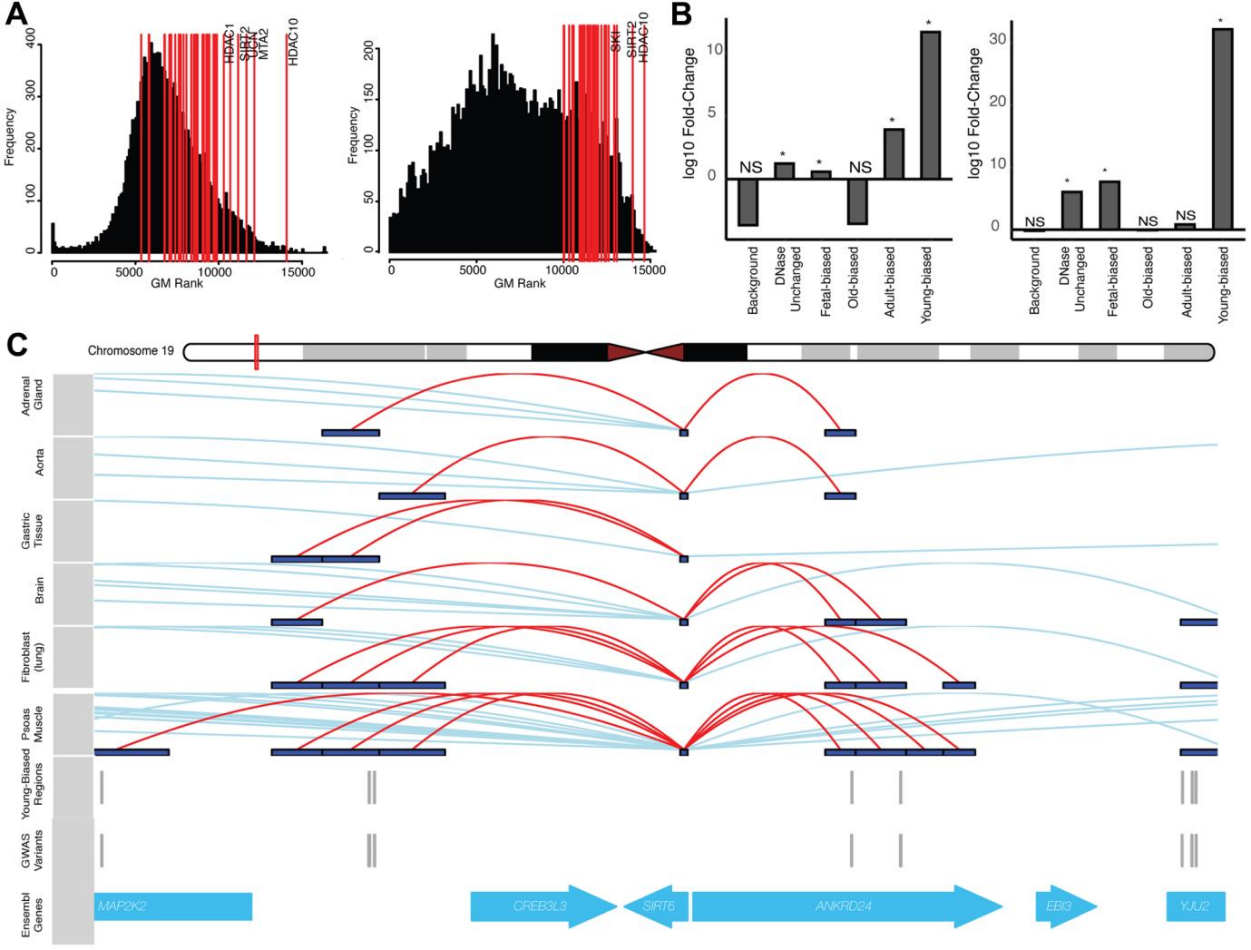
**Altered-accessibility regions identify relevant aging biology.** (**A**) (Left) Distribution of cross-disease ranks for all protein-coding genes, when ranking by local variants independent of accessibility data (see Supplementary Methods). Red lines indicate genes within the ‘histone deacetylation’ (HDAC) GO term; top ranked genes (by geometric mean) are indicated. (Right) Similar distribution of cross-disease ranks, ranking genes with variants nearby young-biased regions. Red lines indicate top HDAC genes by rank. (**B**) (Left) Fold-change of normal cumulative distribution function (CDF) p-values of variants within HDAC gene loci associated with different region sets, relative to CDF test performed using all variants, for cross-disease Z-score metric (see Supplementary Methods). (Right) Similar plot for per-variant LINSIGHT scores. See Supplementary Table 4. (**C**) Variants directly intersecting young-biased regions which interact with the *SIRT6* promoter. (Top) Visualized promoter-capture data [41] across multiple cell-types. (Bottom) Tracks indicating variants which overlap young-biased regions within the *SIRT6* gene locus.

Given our results, found at both genome-wide and gene-locus set levels, we finally sought to take an unbiased approach to identify relevant ‘core’ aging-related genes solely on the basis of aggregate GWAS signals (see Supplementary Methods, Supplementary Information). Overall, we had limited success in defining a set of genes with clear, pan-tissue biological relevance, suggesting that, if such a core does exist, that it may be too broad, or the per-locus signals too moderate, for our method to robustly detect. However, since our results suggest the importance of altered epigenetics in modifying GWAS associations, we performed a similar gene-prioritization analysis using variants occurring nearby altered-accessibility regions (Supplementary Methods). This yielded markedly different enrichments for terms relating to immune processes and gene regulation (see Supplementary Information, Supplementary Table 4). One particular set of genes, involved in histone deacetylation, has repeatedly been linked to aging and epigenetics [61, 62] and was identified using our set of young-age regions (see Figure 4). We explore this set in more detail below.

### Sequence evolution and disease association

Our previous analyses found that patterns of inter- and intra-species sequence conservation depended on epigenetic status (i.e., degree of accessibility) of regulatory elements. Subsequently, we found that the risk association of variants across a number of age-associated diseases also varied based on accessibility change in the vicinity of the variant. Much work has been done on understanding the relationship between sequence conservation and disease risk [63–66]. For example, a transcription factor (TF) binding site may be subjected to negative selection to conserve its sequence and hence function. Mutations that occur within this site would more likely impact *cis*-regulatory biology, and therefore manifest an association with disease. If this disease impacts fitness, then over time, such mutations will be eliminated, so that genetically ‘older’ mutations are less prevalent [65, 67]. Given our interest in the evolution of development and aging processes, we wanted to investigate the role that epigenetics has on this disease-evolution relationship - and whether this holds with our data. By comparing the cross-trait associations of variants falling within and outside primate-conserved sequences (phastCons) [68] (Supplementary Methods), we found that variants within conserved sequences tend to have greater disease associations, along with younger estimated allele age (Supplementary Table 4). These patterns also hold true for phastCons sequences within age- and developmentally-altered regions (Supplementary Table 4), and follow with previously observed enrichments for GWAS associations of conserved, younger (allele age) variants [65, 67, 69].

We next considered primate conservation, estimated allele age, and cross-set association of variants, looking for the effects of nearby accessibility change on these metrics (Supplementary Methods). As an additional metric for predicted fitness consequences, particularly of non-coding variants, we also included per-bp LINSIGHT scores [70], which integrates data on chromatin accessibility, TF binding motifs, and comparative genomics.

First, we found that variants falling near fetal-biased regions were more conserved, younger, and had lower cross-set association, while variants near adult-biased regions behaved oppositely (Figure 3B and Supplementary Table 4). We also found that the predicted functional consequences associated with fetal-biased regions were greater than with adult-biased regions, despite the lower cross-set association with aging-associated diseases (Figure 3C, see Discussion). Variants falling near old-biased regions were less conserved, had older allele age, and had lower cross-set association than their young-biased counterparts (Supplementary Table 4). These old-biased regions were also associated with the lowest predicted functional consequences (in aggregate) of any set, while the set of young-biased regions had the second-highest average. To compare these behaviors with variants of annotated clinical significance we independently examined ClinVar variants, which while demonstrating increased cross-set association, tended to also be more conserved, younger, and have stronger predicted fitness consequences (Supplementary Table 4).

Collectively, our results indicate that variants stratified by nearby accessibility change violate the expected relationship between sequence conservation and disease association (behaviors instead observed for ClinVar variants). Namely, those regions exhibiting the highest sequence constraint (Figure 3C, left) do not also exhibit the strongest aging-disease associations, nor do those regions exhibiting the weakest constraint, as might be expected in a ‘mutation accumulation’ theory of aging [35]. However, when considering predicted functional consequences (LINSIGHT), which are not defined based on aging demographic data, this pattern is reversed (i.e., the most constrained set, fetal-biased regions, had the strongest predicted consequences despite weaker aging-disease associations). This unexpected behavior may have important implications for evolutionary models of late-onset complex disease genetics. Based on our results, we propose such a model suggesting the outsized impact of regulatory sequences active in early adulthood on genetic contributions to aging-associated disease risk (see Figure 3C, Discussion).

Our proposed model suggests that focusing on disease risk loci containing such putative regulatory sequences (i.e., young-biased regions), should implicate sets of genes involved in aging biological processes. Our gene-level GWAS analyses using young-biased regions identified genes involved with histone deacetylation as being more consistently associated with aging-disease GWAS signal, a pattern which was diminished when considering gene-level associations in the absence of this epigenetic information (Figure 4A), and when using other region sets (e.g., old-biased regions) (Figure 4B). Histone deacetylation enzymes have known impacts on epigenetic aging biology [7, 61, 62] and aging diseases [71]. Within our young-biased enriched gene set we identified *SIRT6* and *SIRT7* as having multiple variants falling nearby young-biased regions which contacted their respective gene promoters (Figure 4C). Both these enzymes have been associated with maintaining heterochromatin during aging [72–74]; *SIRT7* decreases expression with age, and antagonizes hMSC epigenetic aging [73], while *SIRT6* loss manifests an aging-like state [75]. It may be possible that decreased accessibility of regulatory regions controlling the expression of these genes are involved in decreases in sirtuin expression and heterochromatin [72].

## DISCUSSION

In this study we sought to describe how changing epigenetic context, defined here as changes to chromatin accessibility over both development and subsequent aging, influences the behavior of evolutionary forces and genetic disease risk at the sequence level. To address this question, we defined genomic regions whose chromatin accessibility consistently shift over the course of development and aging. Our approach to identify epigenetic shifts relies on the observation that chromatin accessibility broadly reflects the regulatory capacity at a given locus [39], though we acknowledge that more subtle epigenetic changes (e.g., post-translational modifications, CpG methylation) may not be well captured by this accessibility-based definition of epigenetic context.

We performed several analyses suggesting that these regions reflect developmental and aging signatures from previous literature, including genomic features (e.g., repeat elements, CpG sites), epigenetic states (e.g., euchromatin/heterochromatin) and histone mark data. Gene sets associated with developmentally-altered regions were enriched for immune system function and cellular proliferation terms, echoing an earlier study of fetal to adult epigenetic changes [21]. Furthermore, we found correspondence between these gene sets and genes whose RNA-seq expression generally shifted between fetal and adult tissues. Incorporating an independent RNA-seq dataset of adult age-stratified tissues we did not observe the same level of correspondence with age-altered regions – it is possible that some aspects of epigenetic aging (e.g., global de-repression [3, 5, 76]) may account for this disconnect, whereby local accessibility changes are less-directly linked to local expression changes. Interestingly, comparing patterns of expression change in our fetal-adult and young-old comparisons, we saw similar gene-set enrichments (i.e., cell-cycling biased towards fetal and younger-age samples, immune responses biasing towards adult and older-age samples), suggesting that the continuation of epigenetic shifts we observed across development and aging (Figure 1D) may be mirrored at the expression level.

Given that epigenetic state impacts the potential regulatory effects of deleterious variants [34], we looked to see if local development and/or ageing changes to epigenetic context impacts the strength of association between variants and aging-associated diseases. While it is possible that a number of these aging diseases share genetic correlations [77], that these variants are associated with multiple age-associated diseases is also a key expectation for the functional relevance of age-altered regions. In other words, it is the change in epigenetic context that modifies the regulatory potential of these variants, and this has direct impacts on individual associations with multiple diseases.

According to the fetal programming model, we would expect that regulatory regions most active during early development, both dictating developmental processes as well as responding to environmental perturbations [9], would have an out-sized impact on the manifestation of adult-onset diseases. This would be evident in the increased associations of nearby variants with heritable risk for these diseases. However, we found that such fetal-biased regions were not those having the greatest impact with regards to aging disease associations, despite having greater predicted fitness consequences – finding instead that fetal-biased regions are depleted for aging disease GWAS signals, and associated more with developmental diseases/traits (Supplementary Table 4). A recent study of fetal chromatin accessibility at the single-cell level similarly found genetic associations with developmental traits (e.g., height) using regions accessible in different cell-types [78]. We suggest that the ‘fetal programming’ of epigenetic status during early development, genome-wide, has a more moderate impact on aging disease biology than has been previously suggested – though we note that certain developmental loci (e.g., Wnt genes) can and do play a role in aging [8, 24].

According to a model wherein epigenetic aging influences the phenotypic effects of regulatory mutations, we would expect that mutations with increased local accessibility in adult tissues, particularly aged adult tissues, would have stronger impacts on aging disease biology in these tissues (reflected in increased association with heritable disease risk). Here, we found that variants gaining nearby accessibility (i.e., adult-biased regions) have stronger associations across a number of aging-related diseases including several kinds of neoplasms, arthritis, and atherosclerosis. This finding suggests that the regulatory effects of deleterious variants may become ‘uncovered’ as tissues mature and follows with proposed links between development and ageing processes [8, 20, 24]. However, we also found that regions most accessible later in life, when these diseases manifest, are actually associated with weaker GWAS variants. This young/old bias in aging-disease GWAS signal was far stronger than the fetal/adult bias (i.e., the young-biased set was more strongly enriched than adult-biased, and vice-versa). Taken together, these results suggest that (1) accessibility changes in aging tissues have a greater effect on aging tissue diseases, but (2) that variants more accessible earlier in adult life play a bigger regulatory role in contributing to disease risk than do those which gain accessibility later on. Disruptions to regulation in younger tissues may act to set tissues down a path of increasing dysfunction and decline, especially if deleterious variants are able to (cumulatively) contribute to dysfunction as they gradually lose activity with age. In other words, by the time an individual reaches old-age their tissues have had sufficient time to accumulate these dysfunctional effects, ‘setting the stage’ for disease manifestation. Variants more active in old-age, by contrast, have less of an impact on disease manifestation, as their regulatory effects have had less time to integrate. It may be that the time at which disease prevention and/or intervention would be most effective is, perhaps non-intuitively, early in adult life rather than once phenotypes manifest.

We cannot rule out the effects of cell-type specific epigenetic (accessibility) shifts influencing the phenotypic impacts of regulatory sequence modifications on aging-associated disease risk. Similarly, it has been suggested that a facet of aging is ‘epigenetic drift’ – the accumulation of epigenomic aberrations that contribute to mis-regulation of gene regulatory networks, a component of which is tissue-specific [79, 80]. However, the pan-signals which we do observe with respect to evolutionary forces, disease associations, and sets of implicated gene loci indicates the relevance of our approach in understanding the broader components of development and aging-accessibility changes, which may be complemented with future research focusing on those more tissue-specific components.

Regulation of general aging-related mechanisms, as well as increases in heritable disease risk, represent phenotypes upon which evolutionary forces may act to modify aging and mortality rates. We found that young-biased regions were enriched for signals of positive selection, a number of which implicated relevant aging-associated genes, and exhibited increased phylogenetic and within-human sequence constraint. Given that these behaviors are intermediate between those observed with regions more accessible in fetal and older-adult tissues, we suggest the following model (Figure 3D).

Regulatory sequences most active during development are subjected to strong negative selection, both to maintain human-derived functional sequences and discourage subsequent modifications, as dysregulation of development would have the largest fitness consequences. Similarly, sequences most active during early adulthood are subjected to negative selection to maintain proper tissue maintenance and discourage disease. However, the strength of this selection is reduced, as we expect fitness benefits/costs to diminish with age as individuals reproduce less frequently [53–55]. Thus, despite the fact that mutations within or nearby these functional sequences stand to have the greatest impact on disease risk (as noted above) they are less efficiently purged, and are allowed to accumulate over generations [35]. Finally, sequences most active in older adults are under relaxed selective pressures and allowed to drift – mutations are permitted and retained, particularly due to the reduced associations that these mutations have with heritable disease risk. Overall, this model suggests that considering the changing epigenetic context of disease-associated variants may help in prioritizing GWAS signals to loci involved in disease biology (e.g., as we saw for histone deacetylases) and, ultimately, the aging processes driving tissue decline and eventual manifestation of aging-associated disease.

## MATERIALS AND METHODS

### Accessibility datasets

DNase-I hypersensitivity datasets were obtained from ENCODE [40] for eight different fetal and adult tissues (adrenal gland, brain, heart, lung, muscle, skin, spleen and stomach) (see Supplementary Table 1 for accessions and metadata). Raw data was processed as described in the Supplementary Methods, with called open-chromatin regions consolidated across replicates and tissues in order to define a final set of reproducible regions. This aggregated set of peaks was then used to assess both pan-tissue, as well as per-tissue, accessibility changes between fetal and adult datasets using the limma package (version 3.46) in R [81, 82]. Differentially-accessible regions were defined using a Benjamini-Hochberg FDR [83] cutoff of < 0.05.

Adult DNase samples were further stratified in order to define age-altered chromatin regions, splitting samples used in the above analysis into those individuals younger than 50 (‘young-adult’) and those older than 50 (‘old-adult’), this age representing a roughly equal split of sample numbers. Not all tissues used in the initial fetal/adult comparison were represented in these age-stratified sets – thus we restricted the tissue comparisons to brain, heart, lung, muscle and stomach tissues. A similar computational method as that used in defining fetal- and adult-biased regions was applied here (see Supplementary Methods). We compared accessibility changes between young and old-adult samples within those regions exhibiting fetal/adult biases, defining young-biased and old-biased regions (again, using an FDR cutoff of < 0.05).

### Promoter accessibility change

All hg19 Refseq gene TSS were obtained from the UCSC Genome Browser [84] and padded 1kb up/downstream to define promoter regions. For each promoter region, DNase read coverage was compared between adult and fetal samples, with resulting data processed using a similar differential-accessibility method as that used above (see Supplementary Methods). Significantly differentially-accessible promoters were defined using an FDR cut-off of 0.05. As an additional, more stringent analysis, we also defined differentially-accessible promoters based on intersections with the above defined region sets (see Supplementary Methods).

Promoter capture datasets: Promoter-capture data was obtained from Jung et al., 2019 [41]; this dataset was generated from promoter-capture assays across a number of different tissues and cell-types. Given our pan-tissue approach, we considered all data (with the exception of OV2, as we excluded sex-specific tissues from all previous obtained datasets). To generate a set of genomic regions which show evidence of contacting gene promoters, we filtered interacting regions to those which contacted their respective promoters in at least two different tissues/cell-types. This moderate filter was used to exclude those regions for which interactions appear to be exclusive to one dataset, while allowing for regions that do not show such exclusivity.

Gene-set enrichment analyses: Gene sets generated in our analyses were tested for enrichment in different GO Biological Process terms using the ‘enrichGO’ function from the clusterProfiler [85] package version 3.16.1, with semantically-similar GO terms collapsed and significantly-enriched terms defined as adjusted p-value < 0.05.

ENCODE RNA-seq datasets: Processed per-gene quantification files, as generated by the ENCODE pipeline were obtained from the ENCODE web portal [40] (see Supplementary Table 2 for file accessions and metadata). Given the limited availability of adult tissue samples for use in differential-expression analysis, we instead defined a less-stringent method to identify broad changes in gene expression which demonstrate consistency across tissues (see Supplementary Methods).

GTEx RNA-seq processing: Processed RNA-seq quantification files were obtained from the GTEx web portal [43] for the following tissues (matching the above young/old-age accessibility comparison): brain (Brain - Cerebellum), heart (Heart – Left Ventricle), lung (Lung), muscle (Muscle - Skeletal) and stomach (Stomach). Similar to the processing performed in Benayoun et al [44], we applied quality filters to remove lowly-expressed and non-coding genes, and subsequently used the same definitions of ‘young-age’ and ‘old-age’ (as in the above analyses) to calculate differential expression using limma-voom (see Supplementary Methods).

Human sequence variation datasets: Variation data from the 1000 Genomes Project phase 3 (1KGP) [86] (n = 2504 individuals) in. vcf.gz format was obtained and intersected with our region sets using tabix [87] (version 1.9) to obtain variants occurring within these altered-accessibility regions. Common variants were defined using a minor allele frequency (MAF) threshold of >= 0.05. These sets of intersected variants were subsequently used to compare sequence variation across region sets, as well as comparing region-intersected variation to genomic backgrounds and feature sets (see Supplementary Methods).

GWAS summary statistics data: To define a set of aging-associated diseases for use in our analyses, we first used broadly-defined categories as described in Chang et al., 2019 [58]. This study described 92 age-related diseases grouped into broader disease categories based on analyses of large-scale demographic datasets. We took these classifications and manually extracted relevant GWAS phenotypes assessed by the UK Biobanks study [57], obtaining pre-processed summary statistics for these phenotypes provided by the Neale lab [77] (https://nealelab.github.io/UKBB_ldsc/downloads.html). These data were subsequently utilized across several bioinformatic analyses (see Supplementary Methods).

Additional computational methods, including implementations of statistical tests described in the Results, are described in detail in the Supplementary Methods document. The datasets supporting the findings of this study are publicly-available – accession codes and URLs are provided in the Supplementary Methods and Tables. Computational code for processing these datasets is available upon reasonable request from the Lead Contact.

## Supplementary Material

Supplementary Information and Methods

Supplementary Figures

Supplementary Table 1

Supplementary Table 2

Supplementary Table 3

Supplementary Table 4
