# Supplementary material for: Shifting epigenetic contexts influence regulatory variation and disease risk
Source: Aging (Albany NY). 2021 Jun 16;13(12):15699–749. doi: 10.18632/aging.203194 (PMC8266365; doi:10.18632/aging.203194)
Supplement: Supplementary Information and Methods [file aging-13-203194-s001.pdf]

SUPPLEMENTARY INFORMATION

Supplementary Information (SI) Text

Comparing altered accessibility regions to genomic annotations, epigenetic states, and additional epigenetic datasets

As past studies have found that altered distribution of certain histone marks (e.g., H3K27ac) are a key feature of fetal to adult epigenetic changes [1–3] as well as epigenetic aging [4], the changes in chromatin accessibility we observe likely also reflects, in part, histone mark modification.

To define the epigenetic context within which our development- and age-altered regions fall, we utilized genome-wide assignments of epigenetic state as defined by the Roadmap Epigenomics Project Consortium [3], which employs a Hidden Markov Model to assign one of several possible epigenetic annotations to 200bp segments of the genome, integrating both chromatin-modification and accessibility datasets to define state probabilities, for different epigenomes (e.g., skin, brain tissues, etc.). Given that our altered regions were defined using a pan-tissue approach, for each 200bp segment we subset those epigenetic states defined for adult tissue samples, and took the state definition recurrent in the majority of samples as an 'adult-majority' assignment (see Supplementary Methods). We next intersected our region sets with these assigned segments, comparing the distribution of regions falling within different epigenetic states to the genome-wide distribution of these states to look for biases (Supplementary Figure 4). Adult-biased regions were enriched for epigenetic states associated with transcription, heterochromatin, and repressed Polycomb regions (Supplementary Table 1). Conversely, fetal-biased regions were enriched for states associated with enhancers, promoters, and 'primary DNase', while also showing a more moderate enrichment for repressed Polycomb regions. Likewise, old-biased regions were enriched for heterochromatin and quiescent states, while young-biased regions were enriched for all other states (Supplementary Table 1). By intersecting the fetal and adult as well as young and old-biased regions, we saw that the enrichments for different fetal and adult sets - i.e., adult-biased with heterochromatic states, fetal-biased with euchromatic states - overrode the young-biased and old-biased enrichment patterns (Supplementary Figure 4). Utilizing publicly-available epigenetic datasets and annotations through the LOLA [5] software (see Supplementary Methods), we again saw overlaps of the adult-biased region set for genomic annotations of 'repressed segments' and repeat sequences in this set, similar to the Roadmap epigenetic state results above (Supplementary Figure 4). Considering fetal-biased regions, we observed enrichments for TSS segments, Promoter/enhancer segments, and Vista enhancers, along with annotated CpG islands. We also saw similar enrichments for young- and old-biased sets (relative to their Roadmap enrichment results), and again saw the overriding fetal and adult patterns of enrichments in intersection sets (Supplementary Figure 5).

We next sought to validate the expected correspondence between development-associated chromatin accessibility and histone modifications, first using an independent dataset of fetal ChIP-seq experiments [1]. This study defined fetal bivalent promoter regions, which are thought to poise expression of developmental genes for rapid induction upon appropriate signaling [6]. Bivalent promoters tended to not be intersected by adult-biased regions, while fetal-biased regions were enriched in these sets ($p < 1e-16$, hypergeometric test, see Supplementary Methods). That these marked promoters responding to developmental signals lose accessibility in adult tissues would be expected [6], suggesting that our approach is capturing signals of epigenetic change in development. As additional validation of correspondence between development-, and potentially age-, associated chromatin accessibility and regions subject to histone modification, we again used LOLA enrichments, along with histone-mark ChIP-seq datasets acquired from primary tissues samples processed by ENCODE [7, 8].

ChIP-seq analyses

Given our use of DNA accessibility datasets, which should reflect the state of local chromatin with respect to chemical modifications increasing/decreasing accessibility, there is an expected concordance between open-chromatin regions defined by DNase-I hypersensitivity and the presence of nearby marks for histone post-translational modifications (i.e., histone ChIP-seq data). To first confirm this expected behavior in our accessibility data obtained from ENCODE, we further obtained ChIP-seq datasets from fetal and adult tissues matching those used in our accessibility analyses (see Supplementary Table 1 for accessions and metadata). Datasets included H3K27ac (an active, euchromatin mark), H3K27me3 and H3K9me3 (facultative and constitutive heterochromatin marks, respectively). Replicable open-chromatin regions in fetal and adult tissues were compared to their respective called ChIP-seq peak datasets looking for adjacency between accessibility and chromatin marks (within 1kb, see Supplementary Methods). For H3K27ac marks in

adult tissues, between 33-82% of replicable DNase peaks in a given tissue had adjacent ChIP-seq peak calls. For H3K27ac in fetal tissues, between 37-71% of replicable DNase peaks had adjacent ChIP-seq peak calls. For H3K27me3 in adult tissues, between 0.6-10% of replicable DNase peaks in a given tissue had adjacent ChIP-seq peak calls. For H3K27me3 in fetal tissues, between 4-27% of replicable DNase peaks had adjacent ChIP-seq peak calls. For H3K9me3 in adult tissues, between 0.05-4% of replicable DNase peaks in a given tissue had adjacent ChIP-seq peak calls. For H3K9me3 in fetal tissues, between 0.19-22% of replicable DNase peaks had adjacent ChIP-seq peak calls. The increased adjacency of DNase regions with H3K27ac (an active mark) compared to H3K27me3 and H3K9me3 (repressive marks) may be expected, given that DNase hypersensitivity should denote more accessible, active regions of chromatin.

We next asked whether the patterns of accessibility change we observed between fetal and adult tissue samples were also evident at the level of histone modifications. We thus applied a similar pipeline to that used in defining altered accessibility to define altered signals for histone marks (using ChIP-seq read coverage as an approximate, continuous metric) (see Supplementary Methods). This resulted in sets of H3K27ac, H3K27me3, and H3K9me3 peaks whose ChIP-seq signal significantly changed across tissues in comparing fetal and adult samples. Conditioning on the above DNase/ChIP-seq adjacency, we first asked whether significantly-DA DNase peaks tended to be adjacent to altered H3K27ac ChIP-seq peaks, above the general expectation for DNase peaks nearby H3K27ac peaks. We observed a 1.21 fold-change (FC) increase in the adjacency of altered DNase and ChIP-seq peaks (hypergeometric test p-value < 1e-16). Given this, we next asked whether, for these adjacent pairs, directionality was shared (i.e., DNase peaks gaining accessibility are adjacent to H3K27ac peaks gaining signal). We found that, of these adjacent pairs, those sharing direction (i.e., adult-biased DNase, adult-biased H3K27ac ChIP-seq) pairs were significantly over-represented (1.72 FC and 1.19 FC for adult/adult-biased and fetal/fetal-biased, respectively, hypergeometric tests comparing overlaps of sets, adjusted p-values < 1e-16).

We similarly checked this adjacency with H3K9me3 peaks changing signal across fetal/adult tissues. We did see a significantly-greater adjacency between significantly-DA DNase peaks and these altered H3K9me3 peaks, above general DNase/H3K9me3 adjacency (1.13 FC increase, hypergeometric test p-value < 1e-16). Of these adjacent pairs, those sharing direction (i.e., adult-biased DNase, adult-biased H3K9me3 ChIP-seq) were significantly under-represented, while those opposing direction were over-represented (1.152 FC and 1.212 FC for adult-biased DNase/fetal-biased H3K9me3 and fetal-biased DNase/adult-biased H3K9me3, respectively, hypergeometric tests comparing overlaps of sets, adjusted p-value < 1e-16). This follows with an expectation that regions gaining constitutive heterochromatic marks should lose local DNA accessibility, and vice-versa.

Next, we considered the adjacency of H3K27me3 changing signal across fetal/adult tissues. We did observe a slight, but significant, increased adjacency between significantly-DA DNase peaks and altered H3K27me3 peaks, above general DNase/H3K27me3 adjacency (1.03 FC increase, hypergeometric test p-value < 1e-16). Of these, those sharing direction (i.e., adult-biased DNase, adult-biased H3K27me3 ChIP-seq) were significantly over-represented (1.20 FC and 1.38 FC for adult/adult-biased and fetal/fetal-biased, respectively, hypergeometric tests comparing overlaps of sets, adjusted p-values < 1e-16).

Finally, we compared adjacent/overlapping (i.e., within 1 kb) developmentally-altered histone signals across different marks. For a given developmentally-altered H3K27ac peak, adjacent H3K27me3 peaks tended to also change (1.24 FC enrichment, hypergeometric test p-value < 1e-16), with regions gaining H3K27ac signal tending to lose adjacent H3K27me3 signal over development and vice-versa (1.59 FC and 1.09 FC for adult-biased H3K27ac/fetal-biased H3K27me3 and fetal-biased H3K27ac/adult-biased H3K27me3, respectively, adjusted p-values < 1e-16 and 1.9e-6, respectively). Comparing adjacent H3K27me3 and H3K9me3 developmentally-altered peaks, we observed opposing patterns, which may reflect their associations with predominantly facultative and constitutive heterochromatin, respectively. Altered H3K27ac and H3K9me3 peaks showed a small but significant degree of adjacency (~3%, 1.19 FC enrichment, hypergeometric test p-value < 1e-16), though the direction change of adjacent peaks were not consistently biased between adult/adult-biased, fetal/fetal-biased, etc., which may reflect the limited number of adjacent pairs (data not shown).

We also considered the LOLA enrichments for external histone-mark datasets, observing that adult-biased regions showed strong enrichments with ChIP-seq datasets for repressive histone modifications H3K36me3, H3K9me3, and H3K27me3 (see Supplementary Figure 5). Conversely, fetal-biased regions showed enrichments for both active (including H3K4me2/3, H3K9ac) and repressive (including H3K9me3 and H3K27me3) histone modifications.

Clock sites analysis

Given the substantial literature on changes in DNA-level methylation across both development and aging, and the observed enrichments for annotated CpG sites in the above LOLA analyses, we next looked for correspondence between our development- and age-altered region sets and CpG sites. In particular, we considered so-called 'clock sites' capable of predicting age across the entire lifespan [9–11]. Firstly, we re-confirmed the enrichment of CpG sites within developmental and age-altered DNase regions using UCSC annotated CpG sites (see Supplementary Methods), then confirmed that this enrichment held for clock sites, observing a small but significant capturing of these sites by developmentally-altered regions (40 of 353 clock sites, p-value < 1e-3 against 1000 randomized region sets). Of these regions, we saw that the fetal-biased set were enriched for overlaps with both clock sites losing methylation with age (hypo-methylated sites) and those gaining methylation with age (hyper-methylated sites), while the adult-biased set was not enriched for either set. We also saw a significant enrichment for clock sites by age-altered regions (16 of 353 clock sites, p-value < 1e-3 against 1000 randomized region sets). Of these, young-biased regions were enriched for overlaps of both hyper- and hypo-methylated clock sites, while we found no overlaps for clock sites with old-biased regions. Finally, we looked for overlaps between clock sites and our region sets at the gene-locus level – clock sites tied with particular genes (e.g., due to falling within promoter or gene-body regions) which overlap gene loci we associated with our region sets (see Supplementary Methods). This yielded significant overlaps for genes associated with developmentally-altered regions (58 genes, hypergeometric p-value = 0.005), though not those associated with age-altered regions (12 genes, hypergeometric p-value = 0.19), and we observed no significant biases in direction sharing (e.g., old-age-associated genes and hypo-methylated regions – chi-sq test p-value > 0.05) (see Supplementary Table 1).

Promoter capture datasets

To better identify biological process whose *cis*-regulatory activity are subject to change we made use of a compendium of promoter-capture Hi-C interactions [12] (see Supplementary Methods) to identify possible promoter contacts made by our region sets. We also sought to incorporate accessibility information for gene promoters (in addition to the regions contacting them), and did this by [1] intersecting gene promoters with adult- or fetal-biased regions, or [2] similar to our treatment of region accessibility changes we also assessed promoter accessibility using DNase-seq read coverage across tissue samples (Supplementary Figure 6, Supplementary Methods). Genome-wide, adult-biased regions tended to have more putative promoter contacts than fetal-biased regions, while old-biased regions tended to have less putative contacts than young-biased regions (zero-hurdle modeling, p-value << 1e-16). Gene promoters gaining accessibility are preferentially contacted by adult-biased regions, with those losing accessibility contacted by more fetal-biased regions than expected (chi-sq test, p < 1e-16), patterns which held when considering young- and old-age accessibility (chi-sq test, p < 1e-16). This bias was also true when considering gene promoter accessibility defined by intersection with our development- and age-altered region sets (see Supplementary Methods). In the context of enhancer-promoter interaction, we observed enrichments in the adult-biased set for gene-ontology terms associated with immune response, sensory perception, and keratinization (Supplementary Table 2). Conversely, fetal-biased sets were enriched for many developmental terms, as well as terms relating to cellular proliferation and TGF-B signaling (Supplementary Table 2). Echoing the fetal-biased enrichments, we found that old-biased regions were weakly enriched (adjusted p-value = 0.037) for chemokine-response terms, as well as sensory perception. However, no significant term enrichments were observed for young-biased regions and promoters.

As an additional means to consider the sets of genomic loci in which our development- and age-altered sets are distributed, we used the GREAT genome-ontology tool (see description of GREAT in Supplementary Methods). Fetal-biased regions were located near genes associated with several developmentally-related terms, such as 'animal organ morphogenesis' and 'embryo development' (Supplementary Table 2). The adult-biased region set yielded enrichments relating to immune processes, such as 'innate immune response' and 'immune effector process', as well as terms related to keratinization (Supplementary Table 2). Young-biased regions were enriched for terms relating to cell-cycling, such as 'mitotic cell cycle process'. Enrichments for old-biased regions were associated with immune processes such as 'regulation of defense response', while also hitting terms related to DNA break repair and 'negative regulation of telomere maintenance' (Supplementary Table 2). Interestingly, when intersecting the fetal/adult and young/old-biased regions we saw a number of additional GREAT terms, while many signals persisted in intersect sets (Supplementary Table 2). For example, adult-biased regions which were also more accessible in older-adult samples were enriched for the 'positive regulation of immune response' term; a signal of post-natal development of immune function would be expected

[13] and that this signal persists into old-age might suggest that we also capture signals of inappropriate immune system behavior (so-called 'inflammaging' [14]).

RNA-seq expression datasets

Given the biological signals we observed by associating our region sets with gene loci, we next looked to see if similar signals are evident with tissue expression datasets. We utilized ENCODE RNA-seq datasets [8] for fetal and adult tissues – however, given the limited availability of adult tissue samples we performed a less-stringent method for identifying genes whose expression changes over development (see Supplementary Methods). These broad sets of genes yielded similar enrichments to those seen previously on the regulatory level, with genes generally less-expressed in adult tissues enriched for terms involved in growth (e.g., cell-cycling) and chromatin regulation, while those generally more-expressed in adult tissues enriched for terms relating to immune response (e.g., 'humoral immune response'), sensory perception and keratinization (Supplementary Table 2). These gene sets significantly overlapped those genes associated with adult-biased and fetal-biased regions (all genes – 1.11 FC enrichment, hypergeometric p-value = 6.73e-10) and tended to share directionality (chi-sq test, p-value < 1e-16).

We performed a similar expression analysis using adult tissue samples, stratified by the same age categories used in our accessibility analyses, for those adult tissues available from the GTEx dataset [15] which overlapped our adult-tissue accessibility datasets (brain, heart, lung, muscle and stomach) (see Supplementary Methods). Genes generally less-expressed in older samples were enriched for terms relating to growth, including cell-cycling, mitochondrial function, and protein synthesis/turnover (Supplementary Table 2).

Conversely, genes generally more-expressed in older samples were enriched for terms relating to development, including terms such as 'ECM organization', 'ossification' and 'angiogenesis'. Whether or not this follows with the suggested role for aberrant mysregulation of developmental pathways in aging biology [16, 17] signalling pathways, is unclear however. Comparing these aging accessibility and expression-defined gene sets we did not observe significant overlaps (hypergeometric test, 1.04 FC, p-value = 0.19); this may be the result of a disconnect between epigenetic dysregulation and expression changes with aging at particular loci.

Finally, we looked for overlaps between gene expression in our fetal/adult and young/old-adult comparisons, finding that genes broadly less-expressed in adult tissues (relative to fetal) are also less expressed in older adult tissues (hypergeometric, p = < 1e-16). While we did not see significant overlap in the adult-biased/old-age-biased expression sets, those genes which did overlap were enriched for immune response terms similar to those seen in the adult-biased set (data not shown).

Divergent sequence intersection enrichments

We took an aggregated set of sequences showing increased divergence along the human lineage [18–23] and intersected these with our region sets. Subsequently, we assigned each intersection to the nearest annotated gene, and asked whether these elements are actually contacted by these nearby genes via the promoter-capture datasets we had previously integrated with our region sets. These intersections, as well as whether the nearest annotated gene shows some contact data for the indicated region, are presented in Supplementary Table 3. We highlight two example loci, one associated with the fetal-biased region set, the other with the young-biased region set (both of these sets showing general enrichments for overlaps with our aggregated sequence-divergence set, see Figure 2B and Supplementary Figure 7 and Supplementary Table 3).

A region losing accessibility in adult tissues (i.e., a 'fetal-biased' region) intersects a human-accelerated region [20] intronic to *FGF1*, a fibroblast growth factor associated with numerous developmental processes as well as tissue repair [24]; this region also has promoter-capture data to suggest contact with the *FGF1* promoter. A region losing accessibility in old-adult tissue intersects a human-accelerated region [20] intronic to the *PKNOX2* gene, and which also has promoter-capture data to suggest contact with the *PKNOX2* promoter. This region lies downstream of the variant rs590211, which has previously been identified in a GWAS of extreme longevity [25, 26].

Comparing sequence diversity between region sets

Given the patterns of our different region sets in terms of the presence of common human sequence variation (relative to genomic backgrounds and other features, see Figure 2C), we directly compared the occurrence of common variants in different sets to one another in humans, chimps and gorillas (Supplementary Table 3). Within humans, fetal-biased regions tended to have far lower variation when compared to every other set, with the exception of young-biased regions (for which the difference was insignificant). Conversely, adult-biased regions had greater variation when compared to every other set, with the exception of old-biased regions

(which had higher variation). Accordingly, old-biased regions tended to have greater variation when compared to young-biased regions. Within both chimpanzees and gorillas these differences between accessibility-altered region sets were similarly observed (Supplementary Table 3).

Developmental trait GWAS

Considering our region sets comparing fetal/adult accessibility changes, we would expect that regions (which may potentially act as regulatory elements) more accessible in fetal tissues may have more of an impact on developmental processes than those regions less accessible in fetal tissues, and vice-versa when considering processes such as tissue homeostasis (e.g., in adult tissues). Therefore, in addition to our focus on aging-associated diseases/traits, we similarly collected a set of developmental traits/disease GWAS to confirm this expected behavior with regards to developmental processes.

We observed that fetal-biased regions trended towards having greater numbers of nearby significance-thresholded SNPs (reported association p-value < 1e-6) compared to a general DNase background set across almost all traits used (with the exception of childhood epilepsy). Significant enrichments (hypergeometric test, adjusted p-value < 0.05) were limited to birthweight [27] and height [28], though this may be due to the larger number of SNPs nearby target/background sets observed with these traits (see Supplementary Table 4).
Conversely, adult-biased regions trended towards having decreased numbers of nearby significance-thresholded SNPs across almost all traits used (with the exception of childhood epilepsy). Significant (hypergeometric test, adjusted p-value < 0.05) depletions were observed for birth length, maternal-effect birth weight, childhood BMI, fetal-effect birth weight, gestational-duration and height (Supplementary Table 4).

Longevity GWAS

Given the patterns of association with our altered-accessibility region sets and aging-associated diseases, we also considered four different GWAS summary-statistics datasets for parental lifespan [29, 30]. Compared to DNase regions generally, we observed that fetal-biased regions were not enriched for nearby significance-thresholded longevity SNPs (and trended slightly towards depletion). By contrast, adult-biased regions were significantly enriched for the nearby presence of such variants (hypergeometric test, adjusted p-value < 0.05). Similar to adult-biased regions, young-biased regions were significantly-enriched for two of the four longevity datasets, trending slightly with a third. Old-biased regions were neither significantly enriched nor depleted for longevity GWAS signals, unlike what was seen for aging-associated diseases in general.

Effect-size distributions

In addition to determining whether or not a given variant can act to significantly impact disease heritability, the epigenetic state of a region may also determine the magnitude of this impact. For those variants falling nearby developmentally-altered regions, we also considered the reported effect size for their respective diseases. We observed 40 diseases for which variants nearby adult-biased regions had significantly greater absolute effect sizes, compared to only 3 diseases for which nearby variants had significantly reduced effect sizes (Supplementary Table 4). Given that lowering significance thresholds can increase the amount of heritable variation explained for a given trait, we also considered the effect size distribution of all variants falling near our region sets. Nearly all diseases had biased distributions, with the majority (106 of 127) having larger absolute effect sizes for adult-biased regions (Supplementary Table 4).

Per-disease enrichment testing

For each GWAS set, we defined single nucleotide polymorphisms (SNPs) with strong association signals (p-value < 1e-6) and looked for the presence of nearby epigenetically-altered regions (Supplementary Methods). We observed that, generally, our accessibility data were enriched for nearby variants (Supplementary Table 4), which is expected given that these data will capture non-coding regulatory elements which are concentrated for GWAS signal [31]. First considering accessibility change between fetal and adult tissues, we found that of this general enrichment adult-biased regions associate with a significant proportion of variants across a majority of diseases, while fetal-biased regions associated with significantly less variants than expected (Supplementary Table 4).

We next considered the effects of age-associated accessibility changes on age-related disease GWAS signals. Unexpectedly, we observed that old-biased regions, unlike adult-biased regions, are actually depleted of nearby strong variants across the majority of age-related diseases, while young-biased regions are enriched for such signals (Supplementary Table 4). Furthermore, we found that for intersections of development and age-altered regions that this age-associated behavior outweighs the earlier development behavior. Of the general enrichment in adult-biased regions, a significant portion of this can be attested to

adult-biased regions which lose accessibility in old-age (i.e., young-biased regions), while adult-biased regions which gain accessibility in old-age are actually depleted for such signals (Supplementary Table 4). Conversely, of the general depletion in fetal-biased regions, an insignificant portion of this can be attested to fetal-biased, old-biased region intersects (hypergeometric test adjusted p-value > 0.05), while those strong variants which do fall nearby fetal-biased regions tend to be concentrated near those regions also considered young-biased (Supplementary Table 4).

Gene set ranking tests

To confirm the behavior of our within-disease gene ranking strategy (see Supplementary Methods), we defined a positive-control gene set which would be expected to be strongly-associated with aging diseases using the GO term 'homeostatic process' (GO:0042592). When compared to randomly-sampled gene sets this set had significantly-increased cross-disease gene rankings (Supplementary Table 4). As a negative control, we took a gene set which would not be expected to be strongly associated with aging diseases, those involved in the development of reproductive structures (GO:0003006). This set did not have significantly-increased cross-disease gene rankings.

When looking at gene sets defined by RNA-seq data, we found that genes generally less expressed in adult tissues (fetal-biased) were enriched for cross-disease GWAS signals, while genes more expressed in adults were actually significantly depleted for such signals (Supplementary Table 4). Gene loci with increased expression in older adult tissues were enriched for GWAS signals, as were loci with decreased older-adult expression – suggesting the possibility that a mixture of genes increasing and decreasing expression over time may additively contribute to aging disease biology. It is worth noting that the fetal-biased (expression) genes significantly overlap with young-biased genes (defined by expression), possibly explaining the shared enrichment for GWAS signals, while adult-biased and old-biased genes (by expression) did not significantly overlap - though this overlap set itself, containing a number of immune-related genes, was enriched for GWAS signals (data not shown).

Cross-disease gene ranking genome-wide

It has been suggested that the highly polygenic nature of complex traits and diseases reflects cumulative regulatory modification to a 'core' set of genes who functions most proximately in relevant biology (i.e., the 'Omnigenic model') [32]. If this is indeed the case, we would expect that, for age-associated diseases across multiple tissues, those genes most involved with general pan-tissue aging processes would represent a 'core' set of genes whose dysregulation contribute to heritable risk across aging-associated diseases. We took an unbiased approach to relevant gene discovery, identifying a putative set of 'core' aging-related genes solely on the basis of aggregate GWAS signals genome-wide (without considering accessibility change) (Supplementary Methods). The resulting set of genes was enriched for terms relating to keratinization, sensory perception of smell, and neuron-related terms (e.g., glutamate receptor signaling) (Supplementary Table 4). We previously observed the former two terms in our region-association analyses, which may suggest that the effects of gene clustering (e.g., clustering of keratin genes, olfactory receptors) may bias our locus ranking method. We note that similar enrichments for these terms in our fetal/adult RNA-seq analyses were observed (Supplementary Table 2), though whether this GWAS signal – expression - accessibility concordance is due to broad changes in accessibility and subsequent transcription in gene clusters is unclear. The fact that we observe consistent enrichments for keratinization and smell perception using the RRA-based method may indicate that this method is particularly sensitive to gene-clustering effects.

Our per-disease GWAS analyses suggested the importance of altered epigenetic state, particularly that which occurs between young/old adult tissues, in considering the risk association of variants with aging-associated diseases. Therefore, we looked for consistent cross-set ranking using variants occurring nearby age-association regions (Supplementary Methods). Again, applying an RRA-based method to different accessibility region sets yielded broadly similar terms relating to keratinization and smell perception. However, when applying a functional gene-set enrichment analysis (FGSEA)-based method, we saw greater differentiation in enrichment results. Ranking genes based on variants nearby fetal-biased regions yielded terms relating to developmental processes (e.g., embryonic development, skeletal system morphogenesis), while considering adult-biased regions again yielded enrichments for keratinization. Young-biased regions yielded enrichments for 'histone deacetylation' (discussed in more detail in main text), as well as terms relating to viral infection (e.g., 'viral gene expression'). Finally, old-biased regions yielded the previously-seen enrichments for smell perception and keratinization, though also including enrichments for immune processes (e.g., 'antibacterial humoral response') and DNA methylation.

Intersection set comparisons

We compared our developmentally-associated and age-associated regions directly, here explicitly comparing

age-associated regions with developmental regions not changing with age as a more stringent contrast (Supplementary Methods). Here we also saw the much-stronger biasing of young/old-biased regions; old-biased regions associating with significantly less cross-trait heritability than fetal-biased, while young-biased regions associated with significantly more heritability than all other sets (Supplementary Table 4). Comparing development and age-altered intersection sets, we found that the strong disparity in GWAS associations between the young -and old-biased region sets outweighed the differences between the fetal- and adult-biased region sets. For example, the young-biased /fetal-biased set had the second-highest average cross-trait association, despite fetal-biased regions generally being associated with weaker GWAS signals in the previous fetal/adult comparison. Conversely, the weaker GWAS signals associated with the old-biased region set outweighed the generally-higher signals of the adult-biased region set, actually having a lower average cross-trait association than fetal-biased regions not significantly changing accessibility in the young/old accessibility analysis (see Supplementary Table 4).

Supplementary Methods

Processing accessibility datasets

DNase-I hypersensitivity datasets were obtained from ENCODE [33] for eight different fetal and adult tissues (adrenal gland, brain, heart, lung, muscle, skin, spleen and stomach), retrieving sorted, duplicate-filtered mapped read files (.bam) via the ENCODE web portal [8] in hg19 format. ENCODE file accession codes and metadata for individual samples are provided in Supplementary Table 1. To define reproducible hypersensitivity sites within each tissue, we applied the IDR statistical test [34] (version 2.0.3). Briefly, the IDR method identifies overlaps in peak calls across pairs of sample replicates by comparing ranked peak lists (using MACS2 q-value) to define a reproducibility score curve. These paired peaks are then assigned a pointwise score based on this curve. Peaks are sorted, with those falling below an "irreproducible discovery rate" (IDR) threshold (here defined as 0.05) are taken as the final reproducible peak set across replicates. For each sample, peaks were called with MACS2 [35] (version 2.1.1.2) using the following parameters: '-f BAMPE --nolambda' and '-f BAM --no-model --shift -100 --extsize 200' for paired-end and single-end experiments, respectively. An IDR threshold of 0.05 was applied, with resulting filtered peaksets combined using the 'bedtools merge' function from bedtools [36] version 2.29.1 in those instances where both single-end and paired-end experiments for a given tissue were obtained and processed separately with MACS2/IDR. Peak sets were pooled across individual tissues for a given set of samples (e.g., fetal IDR peak calls) and subsequently pooled using 'bedtools merge -c 1 -o count', filtering for peaks which were overlapped at least twice (i.e., called in at least two different tissues). Finally, peaks were fixed to a constant size by padding 75bp from the centre of each peak (150bp regions), this size based on the average size of called peaks across different sets. These tissue-consolidated peak sets, defined for adult and fetal samples, were then pooled and merged with 'bedtools merge', fixing the final set of peaks to a constant size of 150bp. DNase read-coverage was then quantified within this peak set using the 'bedcov' function of samtools [37] (version 1.5) for each mapped .bam file initially obtained, resulting in a final matrix of read coverages for all peaks across all tissue samples.

Read coverages were imported into R [38] version 4.0.2 via the limma [39] package version 3.46; coverages were subsequently normalized using the TMM method using the 'calcNormFactors' function from edgeR [40] version 3.32.1. Two different models for comparing differential-accessibility across adult/ fetal samples were used. Firstly, we considered within-tissue differences in accessibility with time (i.e., the interaction between tissue*time). Secondly, we considered across-tissue differences in accessibility with time to by accounting for all tissues simultaneously (i.e., using a model of tissue + time). For both models, we performed a standard limma-based analysis using the functions 'voomWithQualityWeights' (setting normalized = 'none', all others left to defaults), 'lmFit', 'makeContrasts', 'contrasts.fit' and finally 'eBayes'. The final sets of statistics comparing differential-accessibility across all peaks were extracted for individual tissues (using the results from the first model) and across tissues (using results from the second model) using the 'topTable' function, applying a Benjamini-Hochberg [41] FDR correction to define peaks significantly changing accessibility (differentially-accessible, DA) (adj. P-val < 0.05). Subsequently, the peaks defined as DA across tissues with time were compared to those defined as DA within tissues using R, with the resulting intersections visualized using ggplot2 version 2.3.3 and gridExtra version 2.3 as shown in Figure 1C. Per-peak DA statistic results for the cross-tissue fetal/adult comparison are provided in Supplementary Table 1, Sheet 2.

Visualizing genomic distribution of epigenetic change

To visualize the distribution of regions exhibiting altered accessibility across the genome (i.e., the DA

peaks defined above), we defined genome-wide windows using the bedtools 'makewindows' function, then intersected our peak sets via bedtools intersect. The resulting tracks were loaded into R using the rtracklayer [42] package version 1.50. To visualize the density of altered peaks generally, for each chromosome the number of regions (adult- and fetal-biased) falling within windows were summed per-window, and subsequently smoothened using the 'smooth.spline' function in R. We subsequently defined a red/blue colour scale based on these smoothened counts. In addition this general density, we also calculated the difference in the occurrence of adult-/fetal-biased peaks/regions within windows, smoothening these values within a given chromosome. We used the karyoploteR [43] package version 1.16 to plot karyotypes for all chromosomes, plotting the density of DA peak occurrence as a red/blue density bar, while the differences in adult-/fetal-biased peak occurrence were visualized as a curve (with diagonal red line indicating no difference in smoothened values). These plots for the first ten autosomes are shown in Figure 1B, with the full set of autosomes shown in Supplementary Figure 2.

Defining age-altered regions

In order to compare DNase-I accessibility across adult samples, the set of adult samples used in the above analysis was subsequently split into those from individuals younger than 50 ('young-adult') and those older ('old-adult'), this age representing a roughly equal split of sample numbers. Not all tissues used in the initial fetal/adult comparison were represented in these age-stratified sets – thus we restricted the tissue comparisons to brain, heart, lung, muscle and stomach tissues. The read coverage matrix defined above was restricted to just these adult samples. Given our interest in considering accessibility change with age in the context of earlier fetal/adult epigenetic change, we further subset the coverage matrix to consider age-altered accessibility in peaks defined as DA between fetal/adult samples (adj. P-val < 0.05). The resulting matrix was again loaded into R using limma, with the subsequent analyses performed similarly to that described above – considering two different models (within-tissue and across-tissue aging differences) to compare young/old samples. We finally compared within- and across-tissue DA peak definitions using R, though due to the reduced sample sizes for performing the within-tissue comparisons there was limited overlap of significant results despite agreement in direction-of-effect (data not shown). Per-peak DA statistic results for the cross-tissue young/old-adult comparison are provided in Supplementary Table 1, Sheet 3.

Generating accessibility heatmaps

To visualize accessibility across different fetal/adult tissues (as see in Figure 1A), we took the TMM-normalized counts matrix defined above and converted counts to counts-per-million (CPM) using the 'cpm' function from edgeR with the following parameters: 'log = T, prior.count = 3'. This CPM matrix was then subset to those peaks which were significantly DA (adj p. < 0.05). For visualization, we then sorted all peaks by their limma-calculated t-statistic, taking the top 1000 peaks showing the strongest increase/decrease in accessibility (between fetal/adult). Normalized CPM values were averaged across individual replicates for a given tissue, with the resulting matrix finally z-score-normalized (per-peak), and plotted using the ComplexHeatmap [44] package version 2.6.2. A similar method was performed using peak sets defined in the above age-altered region analysis, as shown in Supplementary Figure 3. Additionally, we performed the above analyses for individual replicates of a given tissue (e.g., heart samples), as shown in Supplementary Figure 1.

Comparing development and age-associated changes

Peak sets defined as differentially-accessible in either the fetal/adult, or young-adult/old-adult comparisons were read into R and compared for overlaps visually using the VennDiagram [45] package version 1.6.20, as seen in Figure 1D. The directionality of peak overlaps, i.e., fetal-/adult-biased vs. young/old-biased, were compared using a chi-sq test in base R, the results of which are shown in Supplementary Table 1, Sheet 3.

Assigning epigenetic states to region sets

To define the epigenetic context within which our development- and age-altered regions fall, we utilized genome-wide assignments of epigenetic state as defined by the Roadmap consortium [3]. This employs a Hidden Markov Model to analyze epigenetic data, including chromatin modification (ChIP-seq) and accessibility (DNase-seq) data, for a given sample and assign one of several possible epigenetic states for individual 200bp segments genome-wide. The Roadmap dataset contains several such genome-wide state definitions for different tissue and cell-line samples (e.g., skin, brain, etc.). We downloaded state definitions for the 25 state model, which incorporates imputed data for 12 marks, for 127 reference genomes, subsetting to those obtained from adult tissue samples. For each individual 200bp segment we then considered the assigned epigenetic state of this segment across all samples – given our pan-tissue approach to chromatin accessibility changes, we defined an 'adult-majority' state assignment based on the assigned state recurrent across the majority of samples.

For simplicity, we collapsed down similar definitions (e.g., ‘Active Enhancer 1’ and ‘Active Enhancer 2’ being considered ‘Enhancer’) (see Supplementary Figure 4 for final reduced set of states). Our sets of development- and age-altered regions were subsequently intersected with these genome-wide states using bedtools intersect, counting the number of segments intersected that belonged to different categories. This was done considering the unique numbers of segments (i.e., segments intersected by more than one region were only counted once) – allowing for repeat segment counting did not substantially alter enrichment results (data not shown). Finally, for each epigenetic state we compared the number of segments intersected by a given region set (e.g., old-biased regions) to the total number of segments assigned this state genome-wide using the phyper function from base R. P-values from these hypergeometric tests were adjusted for the number of states tested using the Benjamin-Hochberg method – enrichment/depletion results were similar when considering all 25 epigenetic states (data not shown). Enrichments/depletions for each region set were plotted as logFC values using ggplot2 (see Supplementary Figure 4).

LOLA enrichment analysis

The LOLA software [5] version 1.12 was used to test for significant enrichments of our region sets with publicly-available sets of genomic annotations and epigenetic datasets. For these sets of developmentally-altered regions, we used the set of DNase-I regions used in the initial differential-accessibility analysis (i.e., reproducible peaks pooled from adult and fetal tissues) as the background region set (to account for the possibility of inherent biases of open-chromatin regions towards certain datasets/annotations). To visualize these enrichment results, significant enrichments (defined as calculated q-value < 0.05) were first sorted by odds-ratio values, then filtered to remove similar entries (e.g., replicate datasets for a given histone mark in a given cell-type). Furthermore, given our interest in epigenetic and genomic annotation terms, we further filtered significant results to retain histone-mark datasets and annotations. Of this set, the top 20 terms (by odds-ratio) were plotted using ggplot2. For LOLA enrichments using young- and old-biased region sets, the set of regions used in the initial young/old comparison (i.e., peaks differentially-accessible across the fetal/adult comparison) was used as the background region set.

Comparing developmental epigenetic changes with bivalent developmental promoters

A set of bivalent promoter domains defined by Yan et al. 2016 [1] using ChIP-seq datasets generated from fetal brain, heart and liver samples was obtained and pooled (Supplementary Table 2 of Yan et al.). To establish a genome-wide background for promoters, all hg19 Refseq gene TSS were obtained from the UCSC genome browser [46] and padded 2kb up/downstream, following the definition of promoter regions used in Yan et al. The adult-biased and fetal-biased region sets were subsequently intersected with these promoter regions using bedtools intersect. The number of bivalent promoters intersected by development-altered regions were compared to the total number of promoters intersected using the ‘phyper’ function of base R to test for enrichment/depletion. As an additional validation, we randomly sampled promoter regions genome-wide to match the number of promoter regions intersected by adult/fetal-biased sets, generating a background of 1000 sets of randomized promoters for each. The number of bivalent domains intersecting these randomized sets was compared to the number of bivalent domains intersected by adult/fetal-biased regions using the ‘pnorm’ function in base R, confirming the depleted intersections of adult-biased regions and enriched intersections of fetal-biased regions (data not shown).

Processing ChIP-seq datasets

For our analyses comparing patterns of chromatin accessibility with histone modifications, histone mark ChIP-seq datasets were obtained from ENCODE for H3K27ac, H3K27me3, and H3K9me3 marks. These datasets were obtained from fetal and adult tissue samples for tissues overlapping those used in the DNase accessibility analyses above – see Supplementary Table 1 for accession codes and metadata. ChIP-seq peaks called by the ENCODE pipeline were obtained along with mapped (hg19) bam files. Peak calls from biological replicates for individual tissues were consolidated by requiring that peaks be replicated in 2/3 of samples to be considered replicable for downstream analyses. Overlapping peaks were merged and fixed to constant size of 400bp (for H3K27ac) and 300bp (for H3K9me3 and H3K27me3) (size based on average peak call size in individual replicates) using bedtools. Replicable peaks were then pooled across tissues, and subsequently pooled across fetal/adult samples, overlapping peaks again merged and fixed to a constant size.

In order to address the expected concordance between called DNase-I hypersensitivity sites and the presence of nearby histone ChIP-seq peaks, replicable DNase-I hypersensitivity sites defined for fetal and adult tissues were taken and compared with replicable ChIP-seq peaks looking for adjacency. This was done using the bedtools ‘slop’ function, looking for the presence of ChIP-seq peaks within a 1kb window (centred on each

ChIP-seq peak) of a given DNase peak. Nearby adjacency was checked for matched tissues – e.g., H3K27ac peaks defined in adult muscle tissue were compared with DNase peaks defined in adult muscle tissue, with the percent of called DNase peaks having nearby ChIP-seq peaks calculated.

We then applied a pipeline similar to that described above in the treatment of DNase datasets.

ChIP-seq read-coverage was quantified within the final peak set using the 'bedcov' function of samtools (version 1.5) for each mapped .bam file initially obtained, resulting in a final matrix of read coverages for all peaks across all tissue samples.

Read coverages were imported into R via the limma package; coverages were subsequently normalized using the TMM method using the 'calcNormFactors' function from edgeR. Given the reduced number of ChIP-seq samples available for any given fetal/adult tissue comparison, only pan-tissue defined peaks were used in subsequent comparisons with altered-accessibility regions (results for individual tissue comparison models not shown). We performed a standard limma-based analysis using the functions 'voomWithQualityWeights' (setting normalized = 'none', all others left to defaults), 'lmFit', 'makeContrasts', 'contrasts.fit' and finally 'eBayes'. The final sets of statistics comparing differential-accessibility across all peaks were extracted from the pan-tissue model using the 'topTable' function, applying a Benjamini-Hochberg FDR correction to define peaks significantly changing accessibility (differentially-accessible, DA) (adj. P-val < 0.05).

Methylation-site analysis

The set of methylation sites used in defining the methylation-aging clock from Horvath 2013 [47] were obtained (Additional File 3). These sites were separated into those either increasing or decreasing methylation status with age, with the resulting sets of genomic coordinates lifted-over from hg18 to hg19 using the 'liftOver' utility from UCSC [48]. These sites were then intersected with our sets of development- and age-altered regions using regioneR [49] (version 1.8.1) using the 'permTest' function, generating 1000 randomized region sets as a background using the 'circularRandomizeRegions' option and the 'count.once' flag, with all other options set to defaults. Significance was assessed at $p < 0.05$ (Supplementary Table 1). For hypergeometric testing at the gene-locus level, gene annotations for hyper/hypo-methylated sites (as defined in the Horvath dataset) were intersected with the sets of genes associated with our different region sets (defined as described below under 'Defining Region-Associated Genes'), using the 'phyper' function in base R to compare the numbers of overlaps relative to all genes captured in the promoter-capture datasets used (see below). Directional bias (e.g., old-age-associated genes and hypo-methylated regions) was tested using the 'chisq.test' function in base R.

Promoter accessibility change processing

All hg19 Refseq gene TSS were obtained from the UCSC genome browser [50] and padded 1kb up/downstream to define promoter regions. For each promoter region, DNase read coverage was calculated for all fetal and adult tissue samples using the 'bedcov' function of samtools (version 1.5) for each mapped .bam file initially obtained, resulting in a final matrix of read coverages for all peaks across all tissue samples. A limma-voom analysis to define promoters whose accessibility were significantly different across tissues in the fetal/adult comparison was performed, similar to that described above in our initial DNase-I region analysis. Significance was defined as adjusted p-value < 0.05. The same analysis was performed using age-stratified adult samples in order to define promoter regions changing accessibility across tissues (i.e., age-altered promoter regions). To visualize promoter accessibility across fetal/adult and young/old-age tissues (as seen in Supplementary Figure 6), the promoter read-coverage matrices was treated similar to that described above. Given the large number of promoters defined as differentially-accessible, we also defined a more stringent definition of changing promoter accessibility. We intersected promoter regions with our region sets using bedtools – that is, a promoter intersected by a fetal-biased region was considered a fetal-biased promoter, and similarly for age-altered region intersections.

Promoter contact processing

Promoter-capture data was obtained from Jung et al., 2019 [12], particularly the file 'GSE86189_all_interaction.po.txt.gz' which contains processed information on regions contacting the promoters assayed in this study. This dataset was generated from promoter-capture assays across a number of different tissues and cell-types; given our pan-tissue approach, we considered all data (with the exception of OV2, as we excluded sex-specific tissues from all previous obtained datasets). To generate a set of genomic regions which show evidence of contacting gene promoters, we filtered interacting regions to those which contacted their respective promoters in at least two different tissues/cell-types. This moderate filter was used to exclude those regions for which interactions appear to be exclusive to one dataset, while allowing for regions that do not show such exclusivity. We then intersected

these interacting regions with our sets of altered regions in order to suggest the possible regulatory roles that our sets may have (in terms of regulating possible target genes).

Preferential contacts:

We first tested to see whether adult-biased or fetal-biased regions differed in their tendency to contact gene promoters. Interacting regions were labeled as adult-/fetal-biased based on these intersections, and the numbers of said regions interacting with promoters was tested using hurdle modelling as implemented using the 'hurdle' function from the pscl [51, 52] package in R (version 1.5.2). A binomial model was applied for the initial hurdle/zero-counts step, with the subsequent counts modeling done using a negative binomial regression model. Tukey post-hoc testing was performed using the emmeans package version 1.5.5 in R. A similar analysis was performed using young- and old-biased regions.

To test whether adult- or fetal-biased regions preferentially intersected with promoter-capture regions contacting promoters either gaining or losing accessibility (differential accessibility as defined above, adjusted p-value < 0.05), we used the 'chisq.test' function in base R for both the adult/fetal comparison as well as young/old-age. As a more stringent test, we performed a similar chi-sq test for promoters gaining/losing accessibility as defined by intersections with development- or age-altered regions.

Defining region-associated genes

To associate genes with our region sets to suggest regulatory patterns (e.g., adult-biased regions contacting a promoter with increased accessibility across adult tissues relative to fetal), we took the set of promoters for which differential-accessibility was significant in the fetal/adult (or young/old-age) comparison (adjusted p-value < 0.05) and considered the regions putatively contacting these promoters, as defined above. Given that a given promoter may be contacted by regions both gaining and losing accessibility (e.g., adult- and fetal-biased regions) (there being few genes contacted by exclusively one set of regions – data not shown), a chi-sq test was performed per-promoter to test for significant bias in the number of putatively-contacting regions (i.e., a fetal-biased promoter with a greater proportion of putative fetal-biased region contacts), controlling for the global proportion of putative contacts (given that we observed biases in different region sets for having more putative contacts given the above zero-hurdle modelling analyses). These multiple tests were corrected using a Benjamini-Hochberg correction, with genes showing a significant bias in putative contacts sharing direction with promoter accessibility change retained for subsequent gene-set enrichment analyses (see below, Supplementary Table 2).

GREAT: GREAT [53] takes an input set of genomic regions along with a defined ontology of gene annotations; firstly, it defines regulatory domains for all genes genome-wide, then measures the fraction of the genome covered by the regulatory domains of genes associated with a particular annotation (e.g., 'cartilage development'). These fractions are used as the expectation in a binomial test counting the number of input genomic regions falling within a given set of regulatory domains, which results in the reported significance of association between an input region set and a particular gene ontology term. GREAT also performs a more traditional gene-based hypergeometric test to test for significance of region set-ontology association. The program returns a set of enriched ontologies sorted by the joint rankings of FDR-corrected binomial and hypergeometric tests, as reported here in our Supplementary Tables. For each given set of ontologies (e.g., GO Biological Processes) we took the set of ranked terms and filtered for those having either an FDR-corrected binomial or hypergeometric p-value of < 0.05; this was done as, given our large peak sets, the hypergeometric test can become saturated (hence, the option to show enrichments significant by the region-based binomial with the GREAT online service). The top thirty filtered terms were then subset and are provided in Supplementary Table 1.

Gene-set enrichment analyses

Genes associated with our different region sets (as described above) were tested for enrichment in different GO Biological Process terms using the 'enrichGO' function from the clusterProfiler [54] package version 3.16.1. The background gene set was defined as all genes for which promoter-capture data was available for use in our above region-gene association processing. Semantically-similar enriched GO terms were subsequently collapsed using the 'simplify' function from clusterProfiler, using default settings. The top enriched GO terms (sorted by adjusted p-value) for each region-associated gene set are reported in Supplementary Table 2, limiting to the top twenty significant (adjusted p-value < 0.05) terms. Similar results are shown for gene sets defined using expression datasets (Supplementary Table 2).

ENCODE fetal/adult RNA-seq processing

Processed per-gene quantification files, as generated by the ENCODE pipeline, were obtained from the ENCODE

web portal [8] (see Supplementary Table 2 for file accessions). Given the limited availability of adult tissue samples with which to perform a differential-expression analysis, we instead defined a less-stringent method to look for broad changes in expression of genes across tissues, as follows. For each individual tissue, replicates for adult and fetal samples were collapsed by calculating the geometric mean of expression values for each gene. The difference in average expression for each gene was then calculated, with all expressed genes subsequently ranked by these differences. The gene-set ranks for each individual tissue comparison were then aggregated using the 'aggregateRanks' function from the RobustRankAggreg library version 1.1 [55]. Briefly, this method considers the ranking of genes across multiple conditions, detecting genes that are ranked consistently higher than expected given a null hypothesis of uncorrelated ranked sets by assigning a per-gene significance score. We applied this method to genes ranked based on differences calculated as (adult – fetal) as well as (fetal – adult), defining our final sets of 'broadly adult-biased' and 'broadly fetal-biased' genes using an RRA significance cutoff of < 0.05. Overlaps of these gene sets with those defined above based on our region sets was done in R, testing for significant overlap with the 'phyper' function, as well as biases in the direction of these overlaps (i.e., adult-biased by region-association, adult-biased by RRA RNA-seq) using the 'chisq.test' function.

GTEx young/old-adult RNA-seq processing

The following processed RNA-seq quantification files were obtained from the GTEx web portal [15]: GTEx_Analysis_2017-06-05_v8_RNASeQCv1.1.9_gene_reads.gct, GTEx_Analysis_v8_Annotations_SubjectPhenotypesDS.txt, GTEx_Analysis_v8_Annotations_SampleAttributesDS.txt. The scripting written for processing this initial metadata was modeled after similar code used in a study of age-associated expression changes that also made use of the GTEx dataset [56]. Samples were subset to just those used in the young-age/old-age accessibility comparison; brain (Brain - Cerebellum), heart (Heart – Left Ventricle), lung (Lung), muscle (Muscle - Skeletal) and stomach (Stomach). Similar to Benayoun et al., the set of human protein-coding genes was obtained from UCSC [50] (Homo_sapiens.GRCh38.pep.all.fa) and intersected with the subset GTEx expression matrix. Similarly, we subset the GTEx matrix to include only male individuals, though testing yielded similar sets of differentially-expressed genes when considering both sexes (data not shown), and filtered for samples having genotype data as well as RIN scores >= 5. We used the same definitions for 'young-age' (< 50) and 'old-age' (> 50) as in the above accessibility analyses.

We utilized similar processing steps as those outlined above in our young/old-age accessibility analyses. The subset expression matrix was imported into R version 4.0.2 via the limma package version 3.46, applying a quality filter by requiring that genes have an expression value of at least 1 counts-per-million in at least three different samples. The filtered matrix was then normalized using the TMM method via the 'calcNormFactors' function from edgeR version 3.32.1. Two different models for comparing differential-accessibility across adult/fetal samples were used. Firstly, we considered within-tissue differences in accessibility with time (i.e., the interaction between tissue*time). Secondly, we considered across-tissue differences in accessibility with time to by accounting for all tissues simultaneously (i.e., using a model of tissue + time). For both models, we performed a standard limma-based analysis using the functions 'voomWithQualityWeights' (setting normalized = 'none', all others left to defaults), 'lmFit', 'makeContrasts', 'contrasts.fit' and finally 'eBayes'. The final sets of differential-expression statistics extracted for individual tissues (using the results from the first model) and across tissues (using results from the second model) using the 'topTable' function, applying a Benjamini-Hochberg FDR correction to define genes significantly changing expression (differentially-expressed, DE) (adj. P-val < 0.05). Subsequently, the DE genes defined across tissues with time were compared to those defined as DE within-tissues using R, with the majority (> 60%) of pan-tissue-defined DE genes also considered DE in at least two different tissues (data not shown).

Overlaps of pan-tissue-defined DE genes with those defined above based on our young-/old-biased region sets was done in R, testing for significant overlap with the 'phyper' function, as well as biases in the direction of these overlaps (i.e., old-biased by region-association, old-biased by GTEx RNA-seq) using the 'chisq.test' function.

Human-divergent sequence analyses

We took an aggregated set of sequences showing increased divergence along the human lineage [18–23] (see Supplementary Table 3, Sheet 2) and intersected with our regions sets (e.g., fetal-biased regions), along with ATAC-seq data obtained from a separate adult post-mortem brain tissue datasets [57], as well as a previously-published B-lymphocyte dataset [58], to act as controls. GM12878 ATAC-seq data was obtained from GEO datasets (GSE47753) as raw .fastq files (for 50K samples); reads were subsequently mapped to hg19 using the ATAC-seq processing pipeline described in Richard et al [59], with IDR replication performed for n = 4 replicates. Adult brain open-chromatin regions were

obtained from the Brain Open Chromatin Atlas (BOCA) [57], downloading the file ‘https://bendlj01.u.hpc.mssm.edu/multireg/resources/boca_peaks.zip’. Called peaks were pooled across different cell types using the ‘bedtools merge’ function. To account for differences in set size when performing intersections, the number of intersections for any given region set were calculated as intersections/bp of sequence in said set. A background distribution was made by generating 1000 random region sets consisting of 100,000 regions (based on the general set size of our altered-accessibility sets, again accounting for total bp of sequence in each randomized set) with a constant length of 150bp via the bedtools ‘random’ function. These background sets were subsequently intersected with our set of human-divergent sequences to establish a background distribution of randomized intersection counts/bp. The distribution of intersection/bp values for this background set was assessed using the ‘qqnorm’ (R base, version 4.0.3) and ‘qqPlot’ (car [60] version 3.0.8) functions – no obvious deviations from a normal distribution were observed. Additionally as a more stringent significance test, we utilized the fitdistrplus [61] package (version 1.1.1) to determine a possible alternative distribution to fit the data. The ‘descdist’ function was initially used to assess curve behavior; goodness-of-fit statistics (from the ‘gofstat’ function) for gamma, beta, exponential, and log-normal distributions were subsequently compared, with the beta-distribution subsequently selected (this choice also being appropriate given the fractional nature of the datapoints [62]. Beta distribution parameters (‘shape1’ and ‘shape2’ in the R implementation of ‘pbeta’) were fit using a bootstrap method (‘bootdist’ from ‘fitdistrplus’), with the median parameter estimates from 1000 samples used to define the distribution for significance testing of target set intersections/bp values with ‘pbeta’ (upper-tail p-values). Results were subsequently adjusted using BH correction, along with those obtained using the normal CDF distribution (‘pnorm’ in base R). Regions intersecting human-divergent sequences were associated with the closest annotated TSS with the HOMER (version 4.11) [63] ‘annotatePeaks.pl’ script. Subsequently, these regions were merged with the promoter-capture datasets described above, indicating those regions for which contact data is suggestive of possible interactions with the nearest gene promoter (Supplementary Table 3).

Cross-species sequence conservation within region sets

Per-bp phyloP20ways conservation scores [64] were obtained from the UCSC table browser [50] for the hg19 genome. For a given region, scores were averaged over the length of all bp using the ‘bigWigAverageOverBed’ utility from UCSC [48]. Scores across all regions in different sets were compared using the ‘pairwise.wilcox.test’ function in base R, applying a BH post-hoc correction (see Supplementary Table 3). Similar comparison results were observed when using a broader 100-ways alignment score (data not shown). For visualizing distributions of scores across sets (as shown in Figure 2A), the region-averaged phyloP scores for different sets were plotted using the ‘density’ function in base R with default settings.

The sets of altered regions were also compared to those DNase regions considered in our accessibility analyses which did not significantly change in the fetal/adult comparison to act as a control dataset. Region-averaged values for target and control sets were compared using the ‘t.test’ function in base R for a one-sided comparisons. This was done for developmentally-altered region sets, as well as age-altered region sets (the control for the latter being those regions in the age-accessibility analysis which did not significantly change between young/old-age tissue samples) (see Supplementary Table 3, Sheet 1).

Species diversity patterns within region sets

Zero-hurdle modelling

Variation data from the 1000 Genomes Project phase 3 (1KGP) [65] (n = 2504 individuals) in .vcf.gz format was obtained and intersected with our region sets using tabix [66] (version 1.9) to obtain variants occurring within these altered-accessibility regions. Chimpanzee (n = 25) and gorilla (n = 31) sequence data was similarly obtained via the Great Ape Genome Diversity Project (GADP) [67]. Peak sets were lifted-over from hg19 to hg18 for use with the GADP datasets with the UCSC ‘liftover’ utility and relevant liftover chain file. Resulting subset VCF files were converted to tab format with the following Unix command, using bcftools [68] (version 1.8):

```
bcftools query -f '%CHROM\t%POS\t%ID\t%REF\t%ALT[\t%SAMPLE=%TGT]\n' -o out.vcf in.vcf.
```

Variant data for all region sets were down-sampled to n=25 (with replacement, 5 resamples for gorilla and 200 re-samples for the human set) in order to match sample size for all comparisons based on the least-sampled species (chimp), using a custom R script.

Common variants were defined using a minor allele frequency (MAF) threshold of >= 0.05 for all datasets, filtering tab-formatted files using a custom Python script. Counts data was defined as the number of variants intersecting a given region and were averaged over resampled variant sets (see below). Counts data across apes were then compared within a given region set (e.g., young-age regions) to compare intra-species diversity within sequences. Hurdle modeling was used to test for

significant differences in both total number of sequences containing variants (hurdle) as well as degree of variation between species (counts); implemented using the 'hurdle' function from the pscl [51, 52] package in R (version 1.5.5). A binomial model was applied for the initial hurdle/zero-counts step, with the subsequent counts modelling done using a negative binomial regression model. Tukey post-hoc testing was performed using the emmeans package in R (version 1.4.7) for both hurdle/zero-counts and counts models, with significance assessed at adjusted p-value < 0.05 (Supplementary Table 3). Additionally, region sets were compared to one another (e.g., fetal-biased vs. adult-biased regions) within a given species using the above methods.

In order to look at sequence constraint of our region sets within humans, a background distribution was made by generating 1000 random region sets consisting of 100,000 regions (based on the general set size of our altered-accessibility sets) with a constant length of 150bp via the bedtools 'random' function. These sets were subsequently pooled, sorted, and merged using bedtools, with the resulting bed file used to extract variants from the 1KG3 set with tabix (version 1.9). The pooled set of altered-accessibility regions was also used to extract variants from the 1KG3 set. We also considered intersection sets in this analysis (e.g., young-biased / adult-biased regions, etc.).

Additionally, several genomic features were extracted from the HOMER (version 4.0.4) set of genomic annotations provided with the program, including the following sets: exon, intronic, promoter-TSS, and TTS. Regions from RepeatMasker were also obtained from the UCSC Table Browser. These additional sets were used to extract variants from the 1KG3 set. The resulting files were filtered for duplicate variants and subsequently MAF >= 0.05 with bcftools (version 1.8). Variants falling within particular regions in the random background, target (i.e., altered-accessibility regions), and genomic annotation sets were then extracted using tabix. Variants extracted for each set were counted using vcftools (version 0.1.15) '--counts2 --stdout' arguments. Variant counts were then adjusted to account for the number of bp within a given set. The background distribution of these values was investigated using the 'qqnorm' (R base) and 'qqPlot' (car package) functions to look for visible deviations from normality, for which no obvious deviations were observed. Values were standardized and statistical significance was assessed using a CDF of the standard normal distribution as implemented in the 'pnorm' function in R (version 4.0.3). P-values for significant deviations from the background distribution were corrected for the number of sets (n = 13) tested using a BH correction. Significance was defined as adjusted p < 0.05 (Supplementary Table 3).

Chimpanzee genomic depletion analysis

A similar sequence constraint analysis was also performed for chimpanzees. Altered-accessibility region sets were pooled and lifted-over to hg18 using the 'liftOver' utility; a set of 1,000 randomly-generated region sets, consisting of 100,000 regions (based on the general set size of our altered-accessibility sets) with a constant length of 150bp via the bedtools 'random' function. Randomized sequence sets were subsequently pooled, sorted, and merged using bedtools, with the resulting bed file used to extract variants from the GADP set with tabix. Several genomic features were extracted from chimpanzee HOMER genomic annotations, including the following sets: intronic, promoter-TSS, TTS, and exon. Additionally, RepeatMasker elements called for the panTro4 genome were obtained from the UCSC Table Browser. These additional sets were lifted-over to hg18 (flags as indicated above) and used to extract variants from the GADP. The resulting files were filtered for duplicate variants and subsequently MAF >= 0.05 with bcftools (version 1.8). Variants falling within particular elements in the random background, target, and genomic annotation sets were then extracted using tabix. Variants per-set were counted using vcftools (version 0.1.15) '--counts2 --stdout' arguments. Variant counts were then adjusted to account for the number of bp within a given set. The background distribution of these values was investigated using the 'qqnorm' (R base) and 'qqPlot' (car package) functions to look for visible deviations from normality, for which no obvious deviations were observed. For comparison with the above human analysis, background values were standardized and statistical significance was assessed using a CDF of the standard normal distribution with the 'pnorm' function in base R. P-values for significant deviations from the background distribution were corrected for the number of sets (n = 13) tested using a BH correction. Significance was defined as adjusted p < 0.05 (Supplementary Table 3).

Obtaining and processing GWAS summary statistics data

To define a set of aging-associated diseases for use in our analyses, we first used broadly-defined categories as described in Chang et al., 2019 [69]. This study described 92 age-related diseases grouped into broader disease categories based on analyses of large-scale demographic datasets. We took these diseases and used them as the basis for manually searching the set of ICD10 disease codes, data for which was obtained from https://www.cdc.gov/nchs/icd/icd10cm.htm. We pulled all ICD codes which matched keywords from this set of defined age-related diseases and aggregated them across

different ICD categories (e.g., diseases of the circulatory system, nervous system, etc.).

Pre-processed data files from the UK Biobanks study [70] were obtained from the Neale lab (https://nealelab.github.io/UKBB_ldsc/downloads.html) (via the link https://docs.google.com/spreadsheets/d/1EmwlCYYqkoVKqAS71nNDKoN18PyKwysYUppKTSMvKiM/edit?usp=sharing), for all summary-statistics results in this UKB dataset for which ICD10 codes matched those aggregated above for aging-associated diseases. We further subset these traits to those for which liability-scaled h^2 estimates (based on LDSC analyses previously performed on these data [71], taken from the 'UKBiobanks_2019_heritabilities_per_trait.tsv.gz' file) were positive. This resulted in a final set of 129 different summary-statistics datasets for further processing (see Supplementary Table 4 for file accessions and trait descriptions). For these summary statistics, the per-SNP hg19 coordinates were obtained from https://www.dropbox.com/s/puxks683vb0omeg/variants.tsv.bgz.

Adjacent accessibility region associations – per-disease enrichment testing

For a given disease, we took the summary-level statistics and defined a set of variants having an association p-value less than a given significance threshold (using both 1e-6 and a more stringent 1e-8 cutoff, the latter yielding similar results - data not shown), generating a .bed output of SNPs (hg19 coordinates). Subsequently, for a given altered-accessibility region set (e.g., adult-biased regions), we considered the presence of SNPs nearby these regions – this was done to capture the possible effects of local linkage-disequilibrium, wherein a strongly-associated SNP may not fall immediately within a region, but a nearby proxy SNP (which may be the causal variant for the association signal) does intersect. This was done using the 'window' function in bedtools to consider significance-thresholded SNPs falling within 1000bp of a given region. As a robusticity check, we also performed the following per-disease enrichment tests using only those significance-thresholded SNPs falling immediately within regions, observing similar enrichments for DNase regions relative to genomic backgrounds, as well as altered-accessibility sets relative to all DNase regions (data not shown).

To first test whether the global set of DNase regions used in our accessibility analyses (i.e., all regions defined across all adult and fetal tissues) were enriched for nearby significance-thresholded SNPs, we defined a genomic background set by randomly subsampling 972,073 regions of 150bp size (matching the set-size of the global DNase set) from the hg19 genome using the bedtools 'random' function, generating 1000 sets of randomized backgrounds. These randomized sets were then subsequently used to count for nearby significance-thresholded SNPs (for a given disease/trait) using the bedtools 'window' function. These randomized background counts were assessed using the 'qqnorm' (R base) and 'qqPlot' (car package) functions to look for visible deviations from normality, for which no obvious deviations were observed. Values were standardized and statistical significance was assessed using a CDF of the standard normal distribution as implemented in the 'pnorm' function in R (version 4.0.3). P-values for significant deviations from the background distribution were corrected for the number of traits tested ($n = 129$) tested using a BH correction. Significance was defined as adjusted $p < 0.05$ (Supplementary Table 4).

Similar testing was done for our different accessibility-altered region sets, whereby customized genome-wide background sets were generated, randomized set counts were calculated, and target/background enrichments were performed. After p-value adjusting, we observed enrichment for all region sets across the majority diseases, which follows with the general enrichment for nearby significance-thresholded SNPs of all DNase regions (significant enrichments seen for 119 of 129 diseases – Supplementary Table 4). Thus, to condition on this general DNase-GWAS enrichment we implemented a hypergeometric testing approach. For each disease/trait showing significant enrichment/depletion using all DNase regions, we counted the number of unique regions (in the set, e.g., adult-biased regions) for which nearby significance-thresholded SNPs were observed, comparing this to the number of general DNase regions for which nearby significance-thresholded SNPs were observed (via the 'phyper' function in base R). For each region-set considered, the resulting set of p-values was adjusted for the number of diseases tested ($n = 127$) (Supplementary Table 4). In order to perform hypergeometric tests comparing the GWAS associations of developmental-aging intersection sets (e.g., adult-biased, young-biased regions), we defined the background set for testing as the respective set of developmentally-altered regions (i.e., we compare the occurrence of nearby significance-thresholded SNPs for adult-biased, young-biased regions to their occurrence nearby the adult-biased region set as a whole).

To visualize these hypergeometric test results (Figure 3A), adjusted p-values for hyper-geometric tests done using different region sets (e.g., adult-biased regions) were plotted as a barplot using ggplot2 version 3.3.3. For visualization purposes, significant enrichment results were plotted as positive values, while significant depletion results were plotted as negative values.

Additional developmental trait GWAS processing

We manually searched GWAS summary-statistic datasets for traits associated with fetal/adult development, pulling largely from data assembled by the EGG consortium (http://egg-consortium.org/index.html), as well as using a combination of GWAS Central [72], GWAS Catalog [73] and GWAS ATLAS [74]. Both general developmental traits (e.g., birth weight), as well as traits relating to particular tissues relevant to the tissues used in our accessibility analyses (e.g., stomach, brain) were searched for, with data availability (in terms of sufficiently-powered studies) largely limited to the former. The following datasets were obtained:

Birth weight

- Birthweight [27] ftp://ftp.ebi.ac.uk/pub/databases/gwas/summary_statistics/GCST005001-GCST006000/GCST005146
- Fetal-effect birthweight [75] (http://egg-consortium.org/birth-weight-2019.html)
- Maternal-effect birthweight [75] (http://egg-consortium.org/birth-weight-2019.html)
- Childhood obesity [76] (http://egg-consortium.org/childhood-obesity-2019.html)
- Pubertal growth (PGF + PGM combined) [77] http://egg-consortium.org/pubertal-growth.html
- Gestational duration (fetal genome) [78] http://egg-consortium.org/gestational-duration-2019.html
- Birth length [79] http://egg-consortium.org/birth-length.html
- Gastrointestinal congenital defects [80] http://biobanks.dk/GWAS/MEGA_CIDR_IHPS_summaryStats.txt.gz
- Childhood epilepsy [81] http://www.epigad.org/gwas_ilae2018_16loci/JME_BOLT-LMM_final.gz
- Height [28] https://portals.broadinstitute.org/collaboration/giant/images/6/63/Meta-analysis_Wood_et_al%2BUKBiobank_2018.txt.gz

Similar to our above treatment of UK Biobanks summary statistics, for each study we filtered for variants below a significance threshold of 1e-6. We then counted the occurrence of these sets of variants falling nearby our region-altered sets (using bedtools window as above), and compared this occurrence to that observed when considering all DNase regions using a hypergeometric test. For each region set considered we adjusted the resulting hypergeometric p-values for the number of GWAS datasets tested (n = 10). As before, when considering the age-altered accessibility region sets, the background set was defined as those regions changing accessibility in our developmental accessibility analyses.

Summary statistics for additional developmental traits, such as congenital heart defects, celiac disease, etc., were obtained, however, these studies had few or no significant SNPs at the 1e-6 significance threshold used (data not shown).

Additional longevity GWAS dataset processing:

Longevity GWAS summary statistics were obtained from Timmers et al. 2019 [29] and Pilling et al. 2017 [30], particularly:

Parental lifespan (Timmers et. al) (GWAS Catalog ID: GCST009890)
Parental lifespan (mother's attained age, Pilling et al.) (GWAS Catalog ID: GCST006696)
Parental lifespan (father's attained age, Pilling et al.) (GWAS Catalog ID: GCST006701)
Parental lifespan (combined parental age, Pilling et al.) (GWAS Catalog ID: GCST006697)

Similar to our above treatment of UK Biobanks summary statistics, for each set of summary statistics we filtered for variants below a significance threshold of 1e-6. We then counted the occurrence of these sets of variants falling nearby our region-altered sets (using bedtools window as above), and compared this occurrence to that observed when considering all DNase regions using a hypergeometric test. For each region set considered we adjusted the resulting hypergeometric p-values for the number of GWAS datasets tested (n = 4). As before, when considering the age-altered accessibility region sets, the background set was defined as those regions changing accessibility in our developmental accessibility analyses.

Effect-size distribution of variants

For a given disease, the set of significance-thresholded SNPs falling nearby a given set of accessibility-altered regions were extracted from the summary-statistic data along with their reported effect-size (estimated beta value). In order to compare effect-size distributions of SNPs nearby different region sets (e.g., fetal-biased vs. adult-biased regions), the absolute effect size values for SNPs falling nearby the two sets were compared using a two-tailed non-parametric Wilcoxon rank-sum test via the 'wilcox.test' function in base R. The resulting p-values were corrected for the number of diseases compared (n = 127) using a BH correction (see Supplementary Table 4). This testing was carried out first using significance-thresholded SNPs (association p-value < 1e-6), and subsequently tested using all nearby SNPs (not applying a significance threshold).

Per-SNP definitions

We defined a cross-trait metric of disease association which considers the assigned association p-value between a given SNP and multiple different aging-associated diseases. The UK Biobanks summary statistics datasets provide association statistics across the same set of SNPs, such that directly comparing the association values for a single variant across multiple datasets is possible. We first defined the global set of shared variants reported in the majority of GWAS files (for a final set of 13,789,793 SNPs), filtering out those summary statistics data which did not have information for this shared set. For a given summary statistic file, the association p-values assigned to these shared variants were extracted and subsequently standardized using the 'stats.zscore' function from the 'scipy' package [82] version 1.15.4 in Python 3. This was done across all diseases, with the final set of per-SNP z-scores converted to a matrix. This matrix was subsequently summed per-row using 'awk' to produce a per-SNP summarized z-score metric reflecting cross-disease risk associations, such that SNPs having stronger associations across multiple diseases (standardized within each disease) will have larger summed Z-scores.

Region integration with per-SNP metric

Similar to the above per-disease hyper-geometric testing, we considered the cross-disease association metric of SNPs falling nearby accessiblity-altered region sets using the bedtools 'window' function with a window of 1000bp. As above, we similarly performed a robusticity check to confirm that these results were consistent with those generated when considering only variants falling immediately within regions (data not shown). To compare the behavior of SNPs associated with our developmentally-altered region sets, we aggregated these per-SNP metrics across adult-biased (n = 3,688,911) and fetal-biased (n = 1,977,122) region sets and compared them using the 'aov' function in base R. As additional controls for this analysis, we also considered the per-SNP metrics of variants falling nearby DNase regions not significantly changing accessibility (acting as a DNase control, n = 2,554,671), and finally compared all these region-associated variants to those variants not associated with any nearby DNase regions (acting as a genome-wide, non-regulatory-element control, n = 6,742,487). Tukey post-hoc analysis was performed with the 'TukeyHSD' function in base R (see Supplementary Table 4). To visualize these results (Figure 3B), we used the 'plotmeans' function from gplots version 3.1.1.

To compare our aging-altered region sets to developmentally-altered regions, as well as the behaviors of intersection sets (e.g., adult-biased, young-biased regions), we similarly aggregated per-SNP metrics across all sets and compared them as above. For the comparisons of intersect sets, we used fetal-biased and adult-biased regions which were not intersected with aging-altered regions, rather than the full region sets, while the DNase control regions, as well as genome-wide control set, remained unchanged. For graphical purposes, the comparison was simplified to show age-altered and developmentally-altered region sets separately (Figure 3B).

Comparing cross-set SNP metric with PhastCons

PhastCons [83] 20ways-defined conserved regions were downloaded from the UCSC table browser in hg38 coordinates, and subsequently lifted-over to hg19 with the 'liftOver' tool. We partitioned SNPs genome-wide as those falling within or outside these PhastCons elements, then compared the cross-trait SNP metrics of these two partitions using a two-sided Wilcoxon test using the 'wilcox.test' function in base R. SNPs nearby accessibility-altered region sets were also partitioned based on PhastCons elements to confirm the cross-set metric behavior of subset variants. We also ran these PhastCons comparisons for SNPs subset by different region set, consistently observed an increased cross-set metric for variants falling nearby phastCons elements (Supplementary Table 4).

Integrating additional per-SNP information

phyloP20ways per-nucleotide data was intersected with the global set of variants for which the per-SNP cross-set association metric was calculated to assign a single phyloP20ways score to each variant. Argweaver [84] estimated allele ages, based on the European subset of the 1000 Genomes project [65], were obtained from http://compgen.cshl.edu/ARGweaver/CG_results/download/bigWigs/?C=S;O=A, and assigned to individual variants using the 'bigWigAverageOverBed' utility from UCSC. Variants for which estimated allele ages were not available were excluded from subsequent analyses. Pre-computed LINSIGHT [85] scores were obtained for the hg19 genome from https://github.com/CshlSiepelLab/LINSIGHT. These were similarly assigned to individual variants using the 'bigWigAverageOverBed' utility.

ClinVar variant testing

ClinVar variants were obtained from the UCSC table browser in hg19 coordinates. We intersected this SNP set with the global set of variants (filtered based on integration of additional per-SNP information) for which the per-SNP cross-set association was calculated.

Given the larger number of SNPs not part of the ClinVar set, we subsampled these SNPs to match the number of ClinVar variants used (n = 76778 SNPs), generating 1000 sets of randomized background variants. For each subset the average cross-trait association metric, phyloP20ways, estimated allele age and LINSIGHT score for all variants was calculated. These averages were used as a background set to compare against the average values in the ClinVar set; for each feature the distribution of randomized values was assessed using the 'qqnorm' (R base) and 'qqPlot' (car package) functions to look for visible deviations from normality, for which no obvious deviations were observed for different features. Values were standardized and statistical significance was assessed using a CDF of the standard normal distribution as implemented in the 'pnorm' function in R (version 4.0.3) (see Supplementary Table 4). As an additional robusticity check, the first and third quartiles for all of these values were also used to calculate significant deviations from randomized background values.

Cross-disease gene ranking

All hg19 Refseq gene TSS were obtained from the UCSC genome browser, and filtered for genes with assigned peptide sequences (obtained from the Table Browser as a 'known canonical' gene table) (i.e., protein-coding genes). This gene set was then padded 100kb up/downstream to define 200kb per-gene windows. For a given disease, we considered all variants falling within all gene windows, selecting the strongest-associated variant falling within each and assigning this association p-value to that particular gene. All genes were then ranked according to their assigned association p-values within a given disease.

To test for significant-enrichment of ranks for a particular set of genes (i.e., a set of genes have nearby assigned SNPs that rank them consistently higher across a number of diseases), all protein-coding genes were first considered: counting how often a given gene appeared in the top 75th percentile of ranked protein-coding genes across different diseases (ranging from 1 to 127). The distribution of these counts for the target set of genes was compared to that of the global distribution of protein-coding genes (exclusive of the target set) using a one-sided (alternative = "greater") Student's t-test in base R.

As a positive control for this analysis, genes associated with the GO term 'homeostatic process' (GO:0042592) were used as a target set for testing. As a negative control, genes associated with the GO term 'developmental process involved in reproduction' (GO:0003006) were used as a target set for testing.

Defining 'core' aging genes

These sets of gene rankings were then aggregated using the 'aggregateRanks' function from the RobustRankAggreg [55] library version 1.1. Given that we considered all protein-coding gene loci, we applied a conservative filter to the resulting RRA significance values via the use of a Bonferroni correction – retaining all genes with a corrected value < 0.05. Gene-set enrichment analysis was then performed with the 'enrichGO' function from the clusterProfiler [54] library version 3.16.1, with the background defined as all protein-coding genes used in the gene-ranking analysis. Significant gene-set enrichments were defined as adjusted p-value < 0.05.

In addition to applying this cutoff-based approach to defining highly-ranked gene sets, we also implemented an approach that did not rely on defining a strict cutoff with the RRA method. For a given gene, we took all of the ranks across the different diseases and calculated the geometric mean of ranks. All genes were then sorted based on this final mean-of-ranks, with this ranking used with 'gseGO' function from the clusterProfiler library version 3.16.1 to perform an FGSEA analysis with the following flags: OrgDb = org.Hs.eg.db, ont = "BP", minGSSize = 15, maxGSSize = 500. Significant gene-set enrichments were defined as adjusted p-value < 0.05.

Given our gene-window based method, it is possible that a single strongly-associated variant may be assigned to two or more closely-adjacent genes. We performed a separate ranking analysis collapsing overlapping gene windows, though found that this led to a reduction in the strength of gene-set enrichments of the RRA ranking results (data not shown).

In order to integrate the effects of local accessibility change into these gene-set rankings, the above ranking procedure was done considering only those variants nearby altered-accessibility regions (e.g., young-biased regions) when assigning per-gene association p-values for ranking (Supplementary Table 4).

Characterizing gene-ranking histone-deacetylase enrichments

To visualize the increased average geometric-mean rank of genes associated with histone deacetylation, the set of 'leading edge' genes associated with the GO term 'histone deacetylase' (HDAC) (GO:0016575) from the FGSEA gene-wise ranking analysis (gene set in Supplementary Table 4) was taken and compared with the geometric-mean rank of all other protein-coding genes used in this analysis. This was done for gene-wise

rankings defined when considering all variants falling within a given gene window (Figure 4A, left), as well as rankings defined when considering those variants with nearby young-biased regions falling within a given gene window (Figure 4B, right).

To compare the differences in GWAS signal associations of these HDAC genes when stratifying variants by nearby altered-accessibility regions, we made use of the per-SNP cross-trait association metric defined above.

The sets of gene windows defined for all protein-coding genes were again taken and variants falling within each gene window were collected. Similar to above, variants were binned based on the presence of nearby altered-accessibility regions (e.g., young-biased regions) – rather than considering the strongest variant signal for a given disease and aggregating ranks across, instead the per-SNP cross-trait association metric for variants was used to assign the strongest signal to a given gene window. This was done considering all variants within a window, as well as binned variants, such that a single gene window has multiple assigned values (one per region set used, as well as a region-independent value). These values were assigned for: all variants, variants with no nearby DNase regions (‘Background’), variants with nearby DNase regions not significantly changing accessibility (“DNase Unchanged”), variants with nearby fetal-biased regions (“Fetal-biased”), variants with nearby adult-biased regions (“Adult-biased”), variants with nearby young-biased regions (“Young-Age”), and variants with nearby old-biased regions (“Old-Age”).

The gene-window values for the HDAC gene set were used as target values. The remaining values of all protein-coding genes (exclusive of this target set) was randomly sampled, generating 1000 sets of genes matching the size of the HDAC target set. The seven different types of assigned values (enumerated above) for each gene window were calculated for both target and test sets. For comparing the target and randomized background sets, the average assigned value for each gene set (target and random) was calculated.

For each type of assigned value, the randomized background set values were assessed using the ‘qqnorm’ (R base) and ‘qqPlot’ (car package) functions to look for visible deviations from normality, for which no obvious deviations were observed. Values were standardized and statistical significance was assessed using a CDF of the standard normal distribution as implemented in the ‘pnorm’ function in R (version 4.0.3). To determine whether stronger GWAS variants falling within HDAC gene windows tend to be stratified by nearby altered-accessibility regions (particularly, young-biased regions as suggested by Figure 4A), the enrichment/depletion values for each different type of gene-wise values (relative to their own respective backgrounds) were compared to the enrichment/depletion values calculated when considering all variants (the region-independent value). This was calculated as: -log10((region-specific CDF test p-value) / (region-independent CDF test p-value)), with positive values indicating a stronger deviation from the background distribution when using region-stratified variants when compared to all variants within a given gene window. These values were visualized using ggplot2, as seen in Figure 4B and Supplementary Table 4.

Visualizing promoter-contact datasets

To visualize the interactions between young-biased regions harbouring nearby genetic variants and the *SIRT6* promoter (Figure 4C) we extracted significant promoter-capture interactions (p-value < 0.01) from the *SIRT6* anchor across a subset of cell types representative of our tissue sets (AD2, AO, GA, Hcmerge, IMR90, PO3 and SX, referring to adrenal gland, aorta, gastric tissue, brain, fibroblast (lung), muscle and spleen labels, respectively). These interaction data were visualized using the GenomicInteractions [86] library version 1.24.0.

Supplementary References

1. Yan L, Guo H, Hu B, Li R, Yong J, Zhao Y, Zhi X, Fan X, Guo F, Wang X, Wang W, Wei Y, Wang Y, et al. Epigenomic Landscape of Human Fetal Brain, Heart, and Liver. J Biol Chem. 2016; 291:4386–98. https://doi.org/10.1074/jbc.M115.672931 PMID:26719341

2. Zhu J, Adli M, Zou JY, Verstappen G, Coyne M, Zhang X, Durham T, Miri M, Deshpande V, De Jager PL, Bennett DA, Houmard JA, Muoio DM, et al. Genome-wide chromatin state transitions associated with developmental and environmental cues. Cell. 2013; 152:642–54. https://doi.org/10.1016/j.cell.2012.12.033 PMID:23333102

3. Roadmap Epigenomics Consortium, Kundaje A, Meuleman W, Ernst J, Bilenky M, Yen A, Heravi-Moussavi A, Kheradpour P, Zhang Z, Wang J, Ziller MJ, Amin V, Whitaker JW, Schultz MD, et al. Integrative analysis of 111 reference human epigenomes. Nature. 2015; 518:317–30. https://doi.org/10.1038/nature14248 PMID:25693563

4. Booth LN, Brunet A. The Aging Epigenome. Mol Cell. 2016; 62:728–44. https://doi.org/10.1016/j.molcel.2016.05.013 PMID:27259204

5. Sheffield NC, Bock C. LOLA: enrichment analysis for genomic region sets and regulatory elements in R and Bioconductor. Bioinformatics. 2016; 32:587–89. https://doi.org/10.1093/bioinformatics/btv612 PMID:26508757

6. Voigt P, Tee WW, Reinberg D. A double take on bivalent promoters. Genes Dev. 2013; 27:1318–38. https://doi.org/10.1101/gad.219626.113 PMID:23788621

7. Yue F, Cheng Y, Breschi A, Vierstra J, Wu W, Ryba T, Sandstrom R, Ma Z, Davis C, Pope BD, Shen Y, Pervouchine DD, Djebali S, et al. A comparative encyclopedia of DNA elements in the mouse genome. Nature. 2014; 515:355–64. https://doi.org/10.1038/nature13992 PMID:25409824

8. Davis CA, Hitz BC, Sloan CA, Chan ET, Davidson JM, Gabdank I, Hilton JA, Jain K, Baymuradov UK, Narayanan AK, Onate KC, Graham K, Miyasato SR, et al. The Encyclopedia of DNA elements (ENCODE): data portal update. Nucleic Acids Res. 2018; 46:D794–801. https://doi.org/10.1093/nar/gkx1081 PMID:29126249

9. Horvath S, Raj K. DNA methylation-based biomarkers and the epigenetic clock theory of ageing. Nat Rev Genet. 2018; 19:371–84. https://doi.org/10.1038/s41576-018-0004-3 PMID:29643443

10. Horvath S, Mah V, Lu AT, Woo JS, Choi OW, Jasinska AJ, Riancho JA, Tung S, Coles NS, Braun J, Vinters HV, Coles LS. The cerebellum ages slowly according to the epigenetic clock. Aging (Albany NY). 2015; 7:294–306. https://doi.org/10.18632/aging.100742 PMID:26000617

11. Hannum G, Guinney J, Zhao L, Zhang L, Hughes G, Sadda S, Klotzle B, Bibikova M, Fan JB, Gao Y, Deconde R, Chen M, Rajapakse I, et al. Genome-wide methylation profiles reveal quantitative views of human aging rates. Mol Cell. 2013; 49:359–67. https://doi.org/10.1016/j.molcel.2012.10.016 PMID:23177740

12. Jung I, Schmitt A, Diao Y, Lee AJ, Liu T, Yang D, Tan C, Eom J, Chan M, Chee S, Chiang Z, Kim C, Masliah E, et al. A compendium of promoter-centered long-range chromatin interactions in the human genome. Nat Genet. 2019; 51:1442–49. https://doi.org/10.1038/s41588-019-0494-8 PMID:31501517

13. Georgountzou A, Papadopoulos NG. Postnatal Innate Immune Development: From Birth to Adulthood. Front Immunol. 2017; 8:957. https://doi.org/10.3389/fimmu.2017.00957 PMID:28848557

14. Franceschi C, Garagnani P, Vitale G, Capri M, Salvioli S. Inflammaging and 'Garb-aging'. Trends Endocrinol Metab. 2017; 28:199–212. https://doi.org/10.1016/j.tem.2016.09.005 PMID:27789101

15. GTEx Consortium. The Genotype-Tissue Expression (GTEx) project. Nat Genet. 2013; 45:580–85. https://doi.org/10.1038/ng.2653 PMID:23715323

16. Blagosklonny MV, Hall MN. Growth and aging: a common molecular mechanism. Aging (Albany NY). 2009; 1:357–62. https://doi.org/10.18632/aging.100040 PMID:20157523

17. de Magalhães JP. Programmatic features of aging originating in development: aging mechanisms beyond molecular damage? FASEB J. 2012; 26:4821–26. https://doi.org/10.1096/fj.12-210872 PMID:22964300

18. Bird CP, Stranger BE, Liu M, Thomas DJ, Ingle CE, Beazley C, Miller W, Hurles ME, Dermitzakis ET. Fast-evolving noncoding sequences in the human genome. Genome Biol. 2007; 8:R118. https://doi.org/10.1186/gb-2007-8-6-r118 PMID:17578567

19. Bush EC, Lahn BT. A genome-wide screen for noncoding elements important in primate evolution. BMC Evol Biol. 2008; 8:17. https://doi.org/10.1186/1471-2148-8-17 PMID:18215302

20. Gittelman RM, Hun E, Ay F, Madeoy J, Pennacchio L, Noble WS, Hawkins RD, Akey JM. Comprehensive identification and analysis of human accelerated regulatory DNA. Genome Res. 2015; 25:1245–55. https://doi.org/10.1101/gr.192591.115 PMID:26104583

21. Pollard KS, Salama SR, King B, Kern AD, Dreszer T, Katzman S, Siepel A, Pedersen JS, Bejerano G, Baertsch R, Rosenbloom KR, Kent J, Haussler D. Forces shaping the fastest evolving regions in the human genome. PLoS Genet. 2006; 2:e168. https://doi.org/10.1371/journal.pgen.0020168 PMID:17040131

22. Prabhakar S, Noonan JP, Pääbo S, Rubin EM. Accelerated evolution of conserved noncoding sequences in humans. Science. 2006; 314:786. https://doi.org/10.1126/science.1130738 PMID:17082449

23. Kostka D, Holloway AK, Pollard KS. Developmental Loci Harbor Clusters of Accelerated Regions That Evolved Independently in Ape Lineages. Mol Biol Evol. 2018; 35:2034–45. https://doi.org/10.1093/molbev/msy109 PMID:29897475

24. Ornitz DM, Itoh N. The Fibroblast Growth Factor signaling pathway. Wiley Interdiscip Rev Dev Biol. 2015; 4:215–66. https://doi.org/10.1002/wdev.176 PMID:25772309

25. Sebastiani P, Solovieff N, Dewan AT, Walsh KM, Puca A, Hartley SW, Melista E, Andersen S, Dworkis DA, Wilk JB, Myers RH, Steinberg MH, Montano M, et al. Genetic signatures of exceptional longevity in humans. PLoS One. 2012; 7:e29848. https://doi.org/10.1371/journal.pone.0029848 PMID:22279548

26. de Magalhães JP, Budovsky A, Lehmann G, Costa J, Li Y, Fraifeld V, Church GM. The Human Ageing Genomic Resources: online databases and tools for biogerontologists. Aging Cell. 2009; 8:65–72. https://doi.org/10.1111/j.1474-9726.2008.00442.x PMID:18986374

27. Horikoshi M, Beaumont RN, Day FR, Warrington NM, Kooijman MN, Fernandez-Tajes J, Feenstra B, van Zuydam NR, Gaulton KJ, Grarup N, Bradfield JP, Strachan DP, Li-Gao R, et al. Genome-wide associations for birth weight and correlations with adult disease. Nature. 2016; 538:248–52. https://doi.org/10.1038/nature19806 PMID:27680694

28. Yengo L, Sidorenko J, Kemper KE, Zheng Z, Wood AR, Weedon MN, Frayling TM, Hirschhorn J, Yang J, Visscher PM, and GIANT Consortium. Meta-analysis of genome-wide association studies for height and body mass index in ~700000 individuals of European ancestry. Hum Mol Genet. 2018; 27:3641–49. https://doi.org/10.1093/hmg/ddy271 PMID:30124842

29. Timmers PR, Mounier N, Lall K, Fischer K, Ning Z, Feng X, Bretherick AD, Clark DW, Shen X, Esko T, Kutalik Z, Wilson JF, Joshi PK, and eQTLGen Consortium. Genomics of 1 million parent lifespans implicates novel pathways and common diseases and distinguishes survival chances. Elife. 2019; 8:e39856. https://doi.org/10.7554/eLife.39856 PMID:30642433

30. Pilling LC, Kuo CL, Sicinski K, Tamosauskaite J, Kuchel GA, Harries LW, Herd P, Wallace R, Ferrucci L, Melzer D. Human longevity: 25 genetic loci associated in 389,166 UK biobank participants. Aging (Albany NY). 2017; 9:2504–20. https://doi.org/10.18632/aging.101334 PMID:29227965

31. Klemm SL, Shipony Z, Greenleaf WJ. Chromatin accessibility and the regulatory epigenome. Nat Rev Genet. 2019; 20:207–20. https://doi.org/10.1038/s41576-018-0089-8 PMID:30675018

32. Boyle EA, Li YI, Pritchard JK. An Expanded View of Complex Traits: From Polygenic to Omnigenic. Cell. 2017; 169:1177–86. https://doi.org/10.1016/j.cell.2017.05.038 PMID:28622505

33 Dunham I, Kundaje A, Aldred SF, Collins PJ, Davis CA, Doyle F, Epstein CB, Frietze S, Harrow J, Kaul R, Khatun J, Lajoie BR, Landt SG, et al. ENCODE Project Consortium. An integrated encyclopedia of DNA elements in the human genome. Nature. 2012; 489:57–74. https://doi.org/10.1038/nature11247 PMID:22955616

34. Li Q, Brown JB, Huang H, Bickel PJ. Measuring reproducibility of high-throughput experiments. Ann. Appl. Stat. 5. 2011; 1752–79. https://doi.org/10.1214/11-AOAS466

35. Yang Y, Fear J, Hu J, Haecker I, Zhou L, Renne R, Bloom D, McIntyre LM. Leveraging biological replicates to improve analysis in ChIP-seq experiments. Comput Struct Biotechnol J. 2014; 9:e201401002. https://doi.org/10.5936/csbj.201401002 PMID:24688750

36. Quinlan AR, Hall IM. BEDTools: a flexible suite of utilities for comparing genomic features. Bioinformatics. 2010; 26:841–42. https://doi.org/10.1093/bioinformatics/btq033 PMID:20110278

37. Li H, Handsaker B, Wysoker A, Fennell T, Ruan J, Homer N, Marth G, Abecasis G, Durbin R, and 1000 Genome Project Data Processing Subgroup. The Sequence Alignment/Map format and SAMtools. Bioinformatics. 2009; 25:2078–79. https://doi.org/10.1093/bioinformatics/btp352 PMID:19505943

38. R Development Core Team, R: A Language and Environment for Statistical Computing. 2008. http://www.r-project.org

39. Ritchie ME, Phipson B, Wu D, Hu Y, Law CW, Shi W, Smyth GK. limma powers differential expression analyses for RNA-sequencing and microarray studies. Nucleic Acids Res. 2015; 43:e47. https://doi.org/10.1093/nar/gkv007 PMID:25605792

40. Robinson MD, McCarthy DJ, Smyth GK. edgeR: a Bioconductor package for differential expression analysis of digital gene expression data. Bioinformatics. 2010; 26:139–40. https://doi.org/10.1093/bioinformatics/btp616 PMID:19910308

41. Benjamini Y, Hochberg Y, Controlling the false discovery rate: a practical and powerful approach to multiple testing. J. R. Stat. Soc. Ser. B,. 1995; 289–300.

https://doi.org/10.1111/j.2517-6161.1995.tb02031.x

42. Lawrence M, Gentleman R, Carey V. rtracklayer: an R package for interfacing with genome browsers. Bioinformatics. 2009; 25:1841–42. https://doi.org/10.1093/bioinformatics/btp328 PMID:19468054

43. Gel B, Serra E. karyoploteR: an R/Bioconductor package to plot customizable genomes displaying arbitrary data. Bioinformatics. 2017; 33:3088–90. https://doi.org/10.1093/bioinformatics/btx346 PMID:28575171

44. Gu Z, Eils R, Schlesner M. Complex heatmaps reveal patterns and correlations in multidimensional genomic data. Bioinformatics. 2016; 32:2847–49. https://doi.org/10.1093/bioinformatics/btw313 PMID:27207943

45. Chen H, Boutros PC. VennDiagram: a package for the generation of highly-customizable Venn and Euler diagrams in R. BMC Bioinformatics. 2011; 12:35. https://doi.org/10.1186/1471-2105-12-35 PMID:21269502

46. Kent WJ, Sugnet CW, Furey TS, Roskin KM, Pringle TH, Zahler AM, Haussler D. The human genome browser at UCSC. Genome Res. 2002; 12:996–1006. https://doi.org/10.1101/gr.229102 PMID:12045153

47. Horvath S. DNA methylation age of human tissues and cell types. Genome Biol. 2013; 14:R115. https://doi.org/10.1186/gb-2013-14-10-r115 PMID:24138928

48. Karolchik D, Barber GP, Casper J, Clawson H, Cline MS, Diekhans M, Dreszer TR, Fujita PA, Guruvadoo L, Haeussler M, Harte RA, Heitner S, Hinrichs AS, et al. The UCSC Genome Browser database: 2014 update. Nucleic Acids Res. 2014; 42:D764–70. https://doi.org/10.1093/nar/gkt1168 PMID:24270787

49. Gel B, Díez-Villanueva A, Serra E, Buschbeck M, Peinado MA, Malinverni R. regioneR: an R/Bioconductor package for the association analysis of genomic regions based on permutation tests. Bioinformatics. 2016; 32:289–91. https://doi.org/10.1093/bioinformatics/btv562 PMID:26424858

50. Karolchik D, Hinrichs AS, Furey TS, Roskin KM, Sugnet CW, Haussler D, Kent WJ. The UCSC Table Browser data retrieval tool. Nucleic Acids Res. 2004; 32:D493–96. https://doi.org/10.1093/nar/gkh103 PMID:14681465

51. Jackman S. Classes and Methods for {R} Developed in the Political Science Computational Laboratory. 2017. https://github.com/atahk/pscl/

52. Zeileis A, Kleiber C, Jackman S, Regression Models for Count Data in {R}. J. Stat. Softw. 2008. http://www.jstatsoft.org/v27/i08/

53. McLean CY, Bristor D, Hiller M, Clarke SL, Schaar BT, Lowe CB, Wenger AM, Bejerano G. GREAT improves functional interpretation of cis-regulatory regions. Nat Biotechnol. 2010; 28:495–501. https://doi.org/10.1038/nbt.1630 PMID:20436461

54. Yu G, Wang LG, Han Y, He QY. clusterProfiler: an R package for comparing biological themes among gene clusters. OMICS. 2012; 16:284–87. https://doi.org/10.1089/omi.2011.0118 PMID:22455463

55. Kolde R, Laur S, Adler P, Vilo J. Robust rank aggregation for gene list integration and meta-analysis. Bioinformatics. 2012; 28:573–80. https://doi.org/10.1093/bioinformatics/btr709 PMID:22247279

56. Benayoun BA, Pollina EA, Singh PP, Mahmoudi S, Harel I, Casey KM, Dulken BW, Kundaje A, Brunet A. Remodeling of epigenome and transcriptome landscapes with aging in mice reveals widespread induction of inflammatory responses. Genome Res. 2019; 29:697–709. https://doi.org/10.1101/gr.240093.118 PMID:30858345

57. Fullard JF, Hauberg ME, Bendl J, Egervari G, Cirnaru MD, Reach SM, Motl J, Ehrlich ME, Hurd YL, Roussos P. An atlas of chromatin accessibility in the adult human brain. Genome Res. 2018; 28:1243–52. https://doi.org/10.1101/gr.232488.117 PMID:29945882

58. Buenrostro JD, Giresi PG, Zaba LC, Chang HY, Greenleaf WJ. Transposition of native chromatin for fast and sensitive epigenomic profiling of open chromatin, DNA-binding proteins and nucleosome position. Nat Methods. 2013; 10:1213–18. https://doi.org/10.1038/nmeth.2688 PMID:24097267

59. Richard D, Liu Z, Cao J, Kiapour AM, Willen J, Yarlagadda S, Jagoda E, Kolachalama VB, Sieker JT, Chang GH, Muthuirulan P, Young M, Masson A, et al. Evolutionary Selection and Constraint on Human Knee Chondrocyte Regulation Impacts Osteoarthritis Risk. Cell. 2020; 181:362–81.e28. https://doi.org/10.1016/j.cell.2020.02.057 PMID:32220312

60. Fox J, Weisberg S. An R Companion to Applied Regression, Third Edition. 2019. https://socialsciences.mcmaster.ca/jfox/Books/Companion/

61. Delignette-Muller ML, Dutang C, fitdistrplus : An R

Package for Fitting Distributions. J. Stat. Softw. 2015; 64:1–34. https://doi.org/10.18637/jss.v064.i04

62. Mun J, Advanced analytical models : over 800 models and 300 applications from the Basel II Accord to Wall Street and beyond Wiley 2008. https://dl.acm.org/citation.cfm?id=1386324

63. Heinz S, Benner C, Spann N, Bertolino E, Lin YC, Laslo P, Cheng JX, Murre C, Singh H, Glass CK. Simple combinations of lineage-determining transcription factors prime cis-regulatory elements required for macrophage and B cell identities. Mol Cell. 2010; 38:576–89. https://doi.org/10.1016/j.molcel.2010.05.004 PMID:20513432

64. Pollard KS, Hubisz MJ, Rosenbloom KR, Siepel A. Detection of nonneutral substitution rates on mammalian phylogenies. Genome Res. 2010; 20:110–21. https://doi.org/10.1101/gr.097857.109 PMID:19858363

65. Gibbs RA, Boerwinkle E, Doddapaneni H, Han Y, Korchina V, Kovar C, Lee S, Muzny D, Reid JG, Zhu Y, Wang J, Chang Y, Feng Q, et al. A global reference for human genetic variation. Nature. 2015; 526:68–74. https://doi.org/10.1038/nature15393 PMID: 26432245

66. Li H. Tabix: fast retrieval of sequence features from generic TAB-delimited files. Bioinformatics. 2011; 27:718–19. https://doi.org/10.1093/bioinformatics/btq671 PMID:21208982

67. Prado-Martinez J, Sudmant PH, Kidd JM, Li H, Kelley JL, Lorente-Galdos B, Veeramah KR, Woerner AE, O'Connor TD, Santpere G, Cagan A, Theunert C, Casals F, et al. Great ape genetic diversity and population history. Nature. 2013; 499:471–75. https://doi.org/10.1038/nature12228 PMID:23823723

68. Li H. A statistical framework for SNP calling, mutation discovery, association mapping and population genetical parameter estimation from sequencing data. Bioinformatics. 2011; 27:2987–93. https://doi.org/10.1093/bioinformatics/btr509 PMID:21903627

69. Chang AY, Skirbekk VF, Tyrovolas S, Kassebaum NJ, Dieleman JL. Measuring population ageing: an analysis of the Global Burden of Disease Study 2017. Lancet Public Health. 2019; 4:e159–67. https://doi.org/10.1016/S2468-2667(19)30019-2 PMID:30851869

70. Sudlow C, Gallacher J, Allen N, Beral V, Burton P, Danesh J, Downey P, Elliott P, Green J, Landray M, Liu B, Matthews P, Ong G, et al. UK biobank: an open access resource for identifying the causes of a wide range of complex diseases of middle and old age. PLoS Med. 2015; 12:e1001779. https://doi.org/10.1371/journal.pmed.1001779 PMID:25826379

71. Bulik-Sullivan B, Finucane HK, Anttila V, Gusev A, Day FR, Loh PR, Duncan L, Perry JR, Patterson N, Robinson EB, Daly MJ, Price AL, Neale BM, ReproGen Consortium, Psychiatric Genomics Consortium, and Genetic Consortium for Anorexia Nervosa of the Wellcome Trust Case Control Consortium 3. An atlas of genetic correlations across human diseases and traits. Nat Genet. 2015; 47:1236–41. https://doi.org/10.1038/ng.3406 PMID:26414676

72. Beck T, Shorter T, Brookes AJ. GWAS Central: a comprehensive resource for the discovery and comparison of genotype and phenotype data from genome-wide association studies. Nucleic Acids Res. 2020; 48:D933–40. https://doi.org/10.1093/nar/gkz895 PMID:31612961

73. Buniello A, MacArthur JA, Cerezo M, Harris LW, Hayhurst J, Malangone C, McMahon A, Morales J, Mountjoy E, Sollis E, Suveges D, Vrousgou O, Whetzel PL, et al. The NHGRI-EBI GWAS Catalog of published genome-wide association studies, targeted arrays and summary statistics 2019. Nucleic Acids Res. 2019; 47:D1005–12. https://doi.org/10.1093/nar/gky1120 PMID:30445434

74. Watanabe K, Stringer S, Frei O, Umićević Mirkov M, de Leeuw C, Polderman TJ, van der Sluis S, Andreassen OA, Neale BM, Posthuma D. A global overview of pleiotropy and genetic architecture in complex traits. Nat Genet. 2019; 51:1339–48. https://doi.org/10.1038/s41588-019-0481-0 PMID:31427789

75. Warrington NM, Beaumont RN, Horikoshi M, Day FR, Helgeland Ø, Laurin C, Bacelis J, Peng S, Hao K, Feenstra B, Wood AR, Mahajan A, Tyrrell J, et al. Maternal and fetal genetic effects on birth weight and their relevance to cardio-metabolic risk factors. Nat Genet. 2019; 51:804–14. https://doi.org/10.1038/s41588-019-0403-1 PMID:31043758

76. Bradfield JP, Vogelezang S, Felix JF, Chesi A, Helgeland Ø, Horikoshi M, Karhunen V, Lowry E, Cousminer DL, Ahluwalia TS, Thiering E, Boh ET, Zafarmand MH, et al. A trans-ancestral meta-analysis of genome-wide association studies reveals loci associated with childhood obesity. Hum Mol Genet. 2019; 28:3327–38. https://doi.org/10.1093/hmg/ddz161 PMID:31504550

77. Cousminer DL, Berry DJ, Timpson NJ, Ang W, Thiering E, Byrne EM, Taal HR, Huikari V, Bradfield JP, Kerkhof M, Groen-Blokhuis MM, Kreiner-Møller E, Marinelli M, et al. Genome-wide association and longitudinal analyses reveal genetic loci linking pubertal height growth, pubertal timing and childhood adiposity. Hum Mol Genet. 2013; 22:2735–47. https://doi.org/10.1093/hmg/ddt104 PMID:23449627

78. Liu X, Helenius D, Skotte L, Beaumont RN, Wielscher M, Geller F, Juodakis J, Mahajan A, Bradfield JP, Lin FTJ, Vogelezang S, Bustamante M, Ahluwalia TS, et al. Variants in the fetal genome near pro-inflammatory cytokine genes on 2q13 associate with gestational duration. Nat Commun. 2019; 10:3927. https://doi.org/10.1038/s41467-019-11881-8 PMID:31477735

79. van der Valk RJ, Kreiner-Møller E, Kooijman MN, Guxens M, Stergiakouli E, Sääf A, Bradfield JP, Geller F, Hayes MG, Cousminer DL, Körner A, Thiering E, Curtin JA, et al. A novel common variant in DCST2 is associated with length in early life and height in adulthood. Hum Mol Genet. 2015; 24:1155–68. https://doi.org/10.1093/hmg/ddu510 PMID:25281659

80. Fadista J, Skotte L, Geller F, Bybjerg-Grauholm J, Gørtz S, Romitti PA, Caggana M, Kay DM, Matsson H, Boyd HA, Hougaard DM, Nordenskjöld A, Mills JL, et al. Genome-wide meta-analysis identifies BARX1 and EML4-MTA3 as new loci associated with infantile hypertrophic pyloric stenosis. Hum Mol Genet. 2019; 28:332–40. https://doi.org/10.1093/hmg/ddy347 PMID:30281099

81. International League Against Epilepsy Consortium on Complex Epilepsies. Genome-wide mega-analysis identifies 16 loci and highlights diverse biological mechanisms in the common epilepsies. Nat Commun. 2018; 9:5269. https://doi.org/10.1038/s41467-018-07524-z PMID:30531953

82. Virtanen P, Gommers R, Oliphant TE, Haberland M, Reddy T, Cournapeau D, Burovski E, Peterson P, Weckesser W, Bright J, van der Walt SJ, Brett M, Wilson J, et al. SciPy 1.0: fundamental algorithms for scientific computing in Python. Nat Methods. 2020; 17:261–72. https://doi.org/10.1038/s41592-019-0686-2 PMID:32015543

83. Siepel A, Bejerano G, Pedersen JS, Hinrichs AS, Hou M, Rosenbloom K, Clawson H, Spieth J, Hillier LW, Richards S, Weinstock GM, Wilson RK, Gibbs RA, et al. Evolutionarily conserved elements in vertebrate, insect, worm, and yeast genomes. Genome Res. 2005; 15:1034–50. https://doi.org/10.1101/gr.3715005 PMID:16024819

84. Rasmussen MD, Hubisz MJ, Gronau I, Siepel A. Genome-wide inference of ancestral recombination graphs. PLoS Genet. 2014; 10:e1004342. https://doi.org/10.1371/journal.pgen.1004342 PMID:24831947

85. Huang YF, Gulko B, Siepel A. Fast, scalable prediction of deleterious noncoding variants from functional and population genomic data. Nat Genet. 2017; 49:618–24. https://doi.org/10.1038/ng.3810 PMID:28288115

86. Harmston N, Ing-Simmons E, Perry M, Barešić A, Lenhard B. GenomicInteractions: An R/Bioconductor package for manipulating and investigating chromatin interaction data. BMC Genomics. 2015; 16:963. https://doi.org/10.1186/s12864-015-2140-x PMID:26576536
