# Supplementary material for: Shifting epigenetic contexts influence regulatory variation and disease risk
Source: Aging (Albany NY). 2021 Jun 16;13(12):15699–749. doi: 10.18632/aging.203194 (PMC8266365; doi:10.18632/aging.203194)
Supplement: Supplementary Figures [file aging-13-203194-s002.pdf]

Supplementary Figures

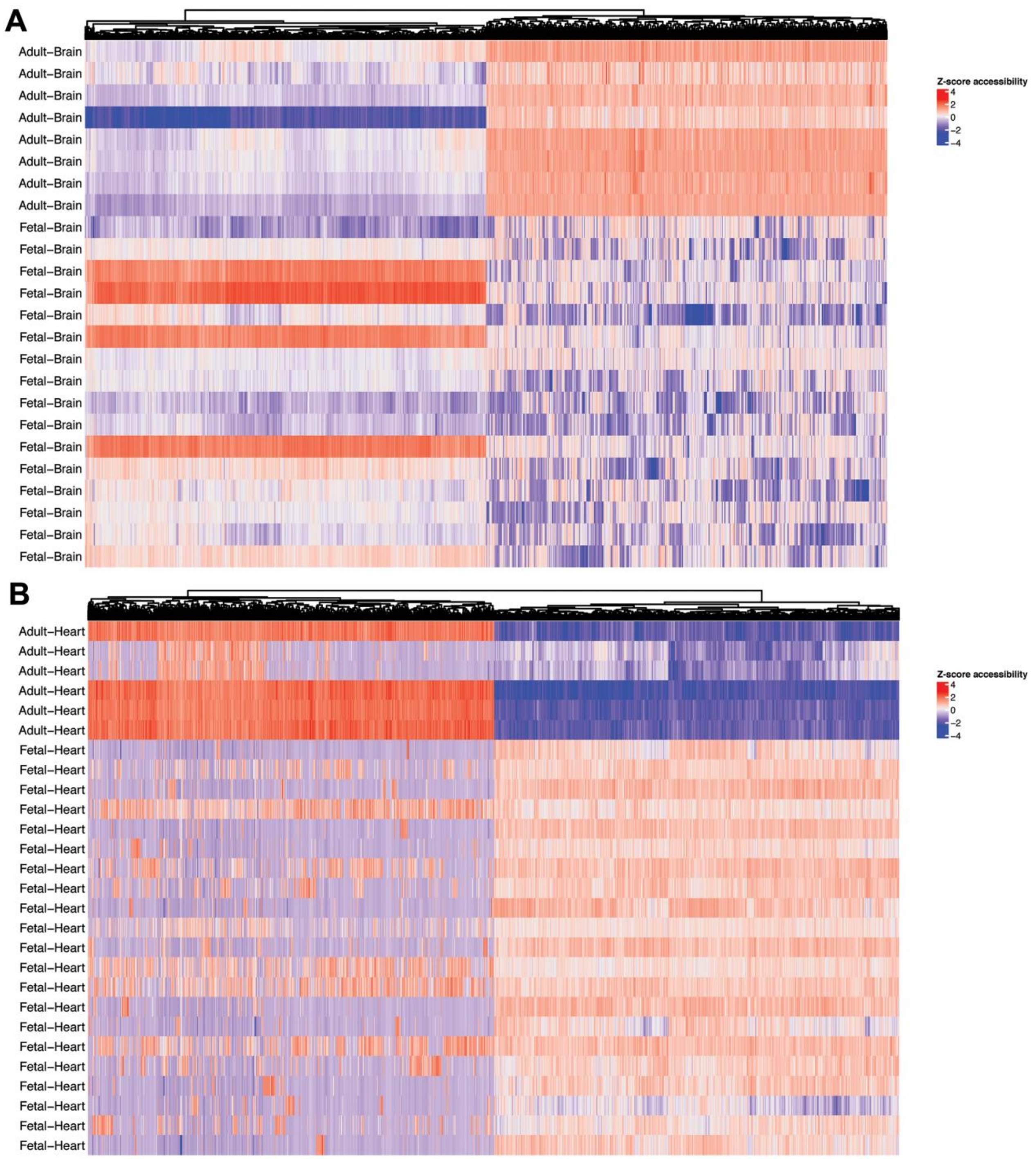

Supplementary Figure 1. Per-tissue heatmaps for brain and heart tissue. (**A**) Z-score accessibility values for regions defined as differentially-accessible comparing fetal and adult brain tissue samples. (**B**) Z-score accessibility values for regions defined as differentially-accessible comparing fetal and adult heart tissue samples. Red-blue colour scale indicates increased/decreased accessibility, z-score normalized per-column.

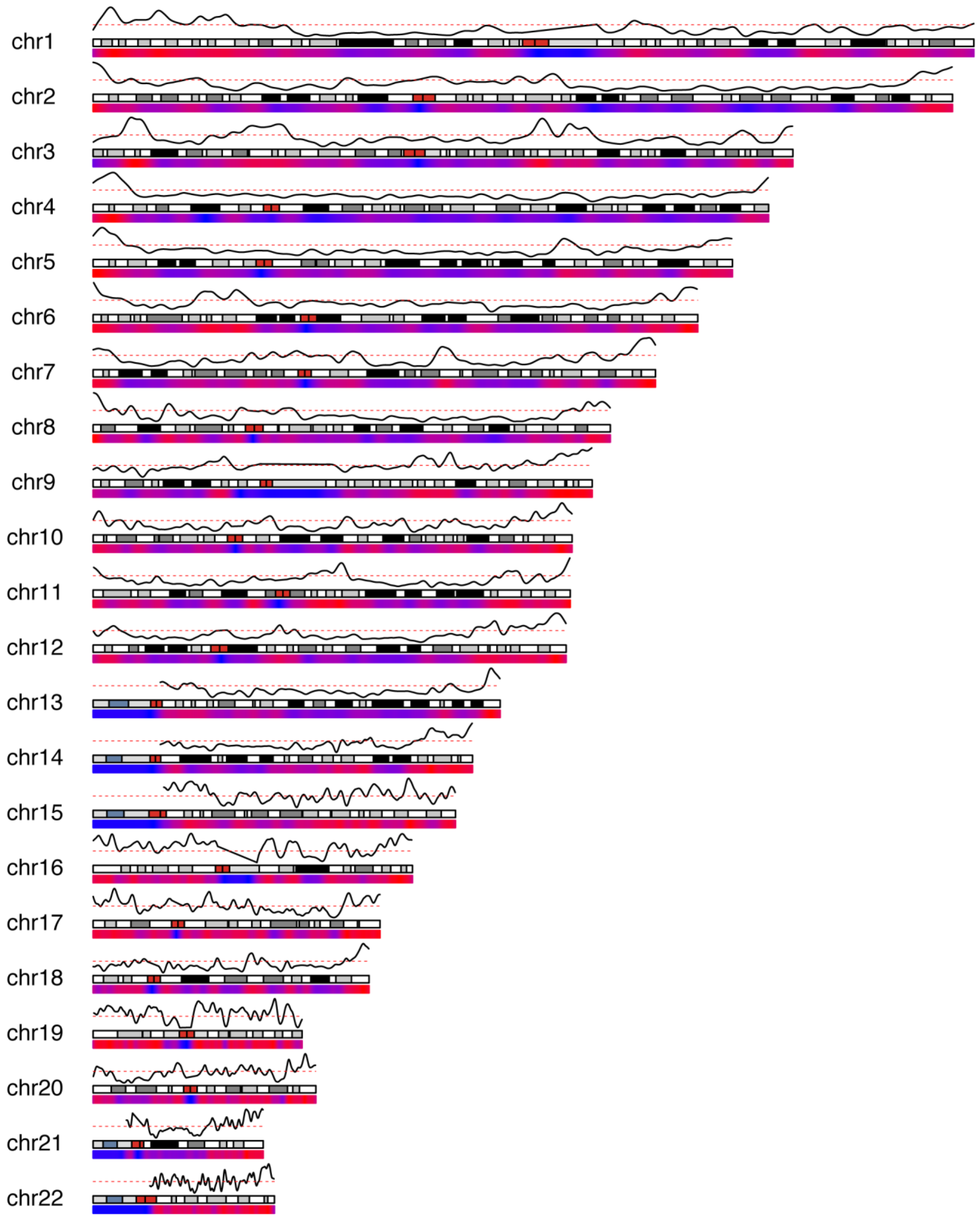

Supplementary Figure 2. Autosome distribution of accessibility-altered regions. Genomic distribution of regions changing accessibility in fetal/adult comparison. Red/blue: density of defined differentially-accessible regions. Line: relative proportion of regions more accessible in adult (top) or fetal (bottom) tissues.

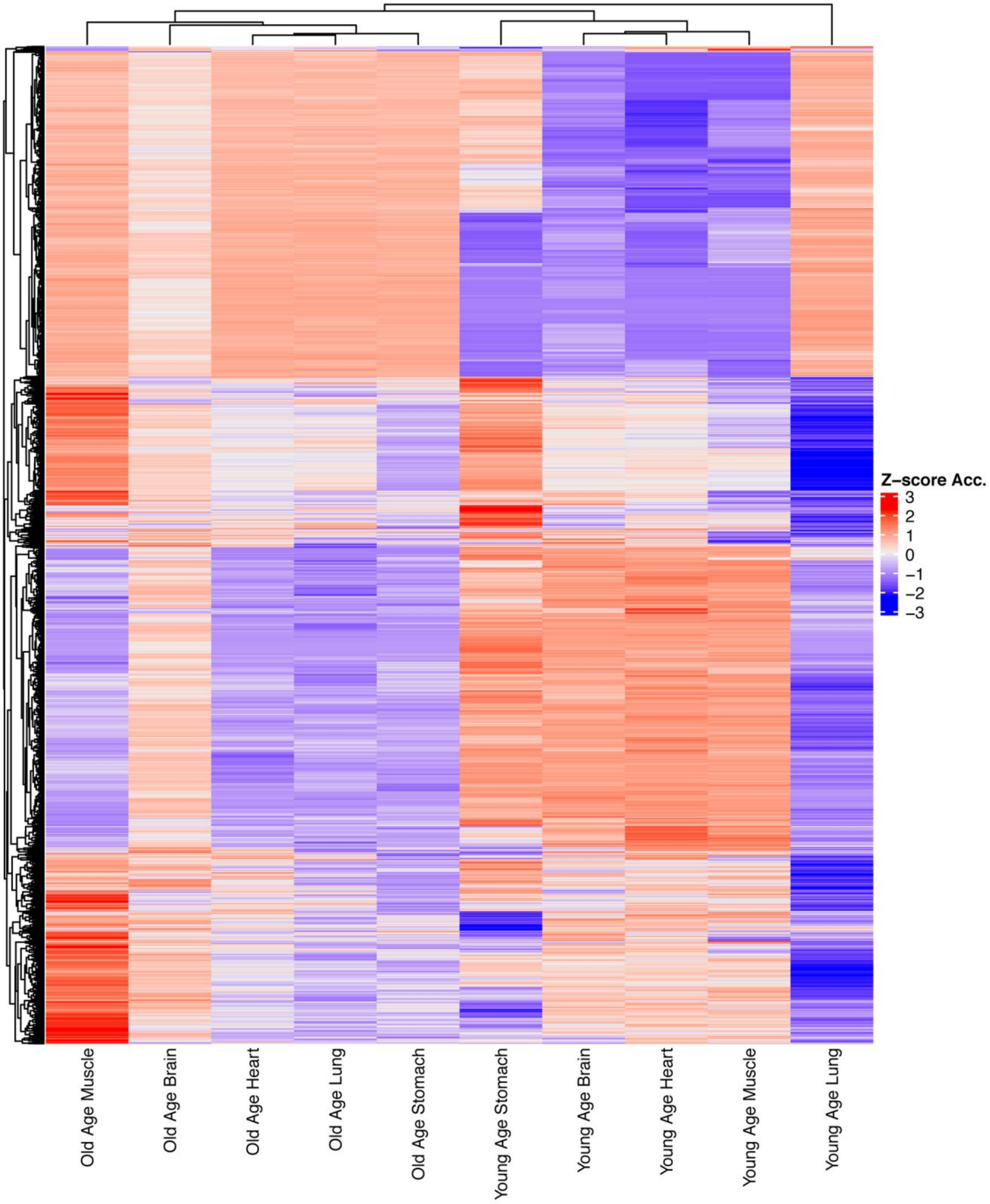

Supplementary Figure 3. Changes in regional accessibility across young-age and old-age adult tissue samples. Equivalent to Figure 1A. Red-blue colour scale indicates increased/decreased accessibility, z-score normalized per-column.

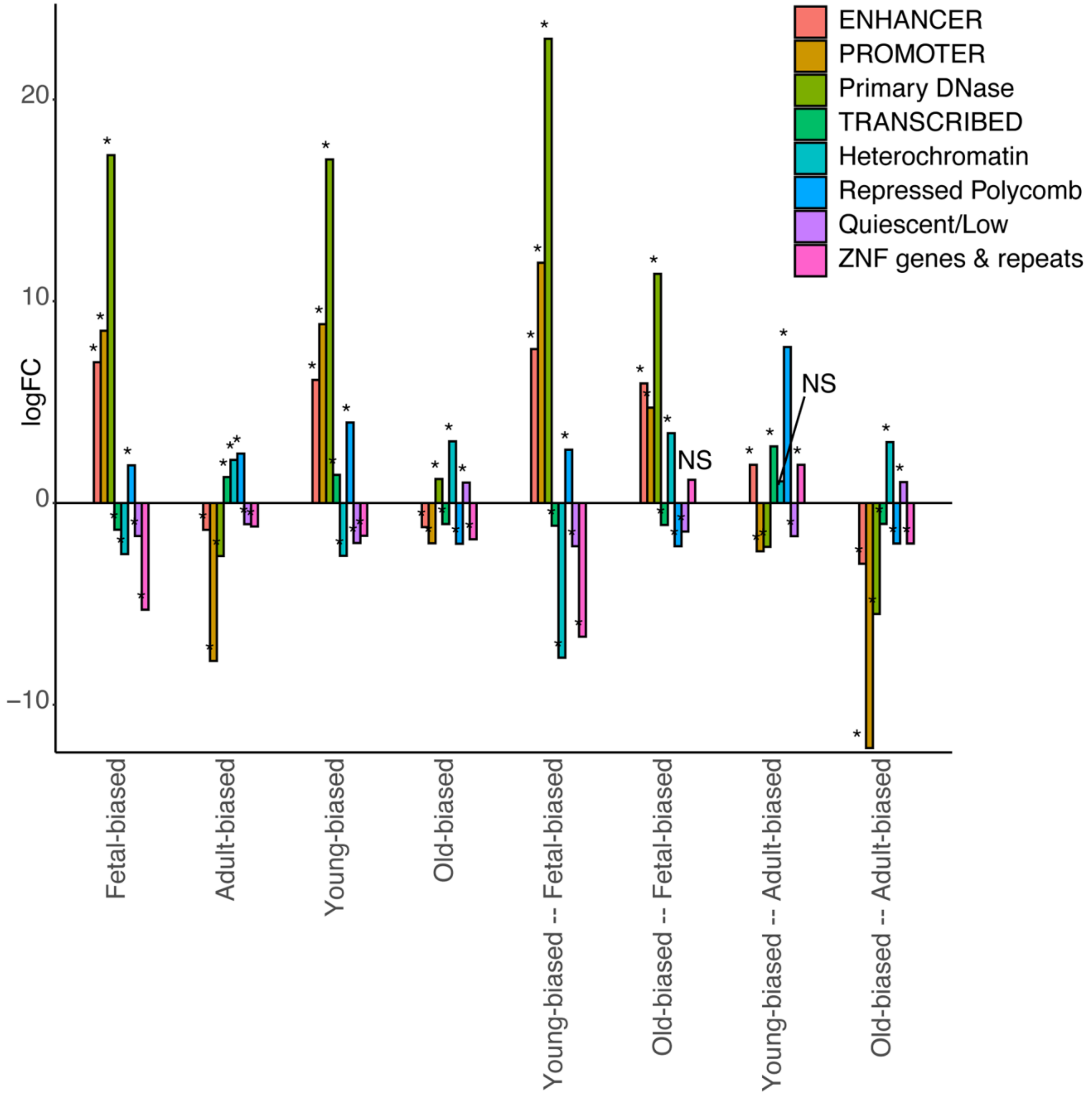

Supplementary Figure 4. Enrichment for epigenetic states. Bar plots indicate log10 logFC enrichment/depletion values for different region sets (e.g., young-biased regions) falling within different Roadmap HMM-annotated epigenetic states. Asterik (*) indicates significant hypergeometric test for enrichment/depletion of an indicated region set for indicated epigenetic state ($p < 0.05$). Intersection sets (e.g., young-biased, fetal-biased intersected regions) are indicated with "—".

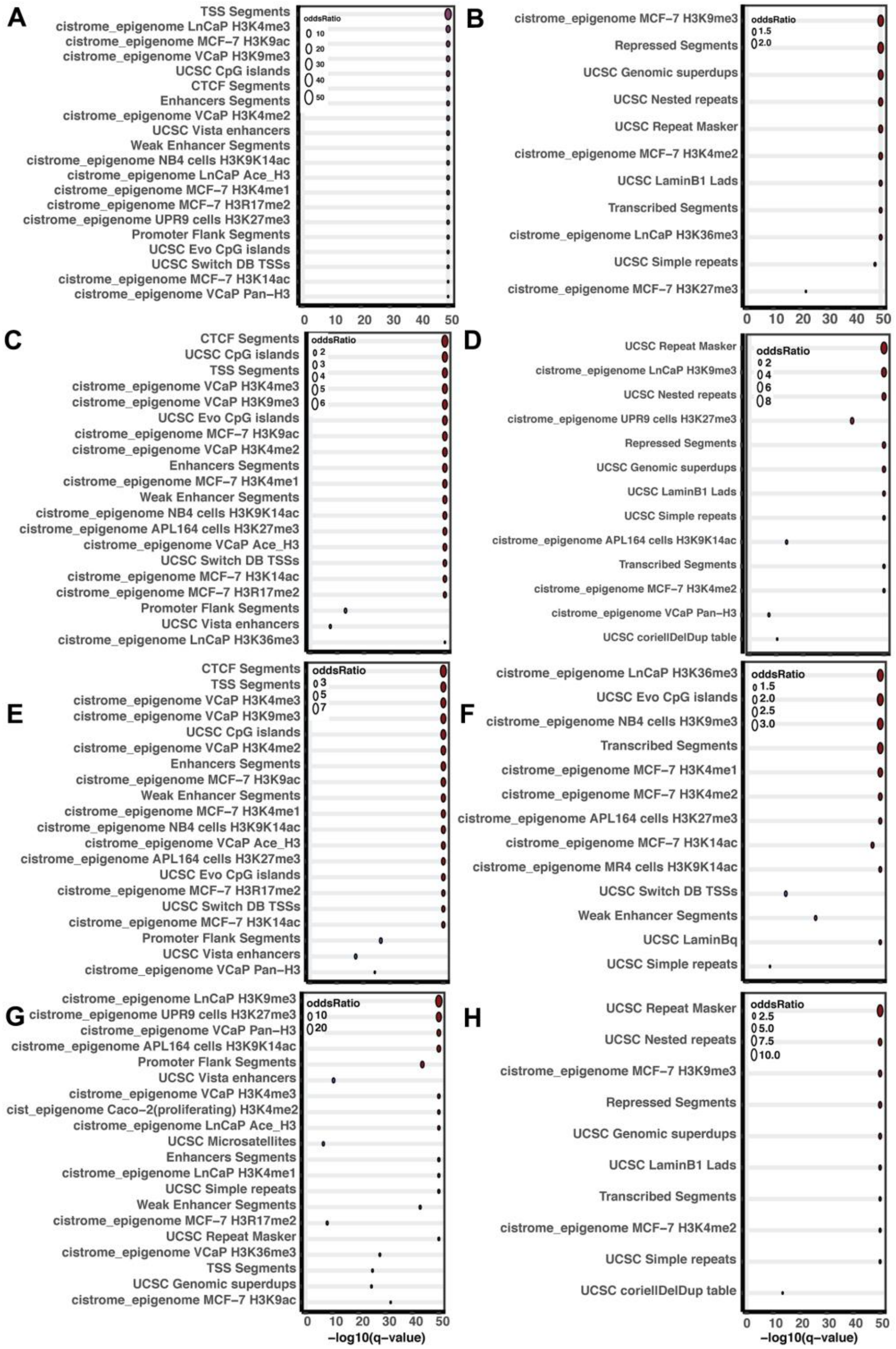

Supplementary Figure 5. LOLA enrichment plots. Enrichment q-values for top terms in the LOLA regional-enrichment analyses. (**A**) Fetal-biased Regions (**B**) Adult-biased Regions (**C**) Young-biased Regions (**D**) Old-biased Regions (**E**) Young-biased – Fetal-biased Regions (**F**) Young-biased– Adult-biased Regions (**G**) Old-biased – Fetal-biased Regions (**H**) Old-biased – Adult-biased Regions. All listed terms are significant, q-value < 0.05. See also Supplementary Table 2.

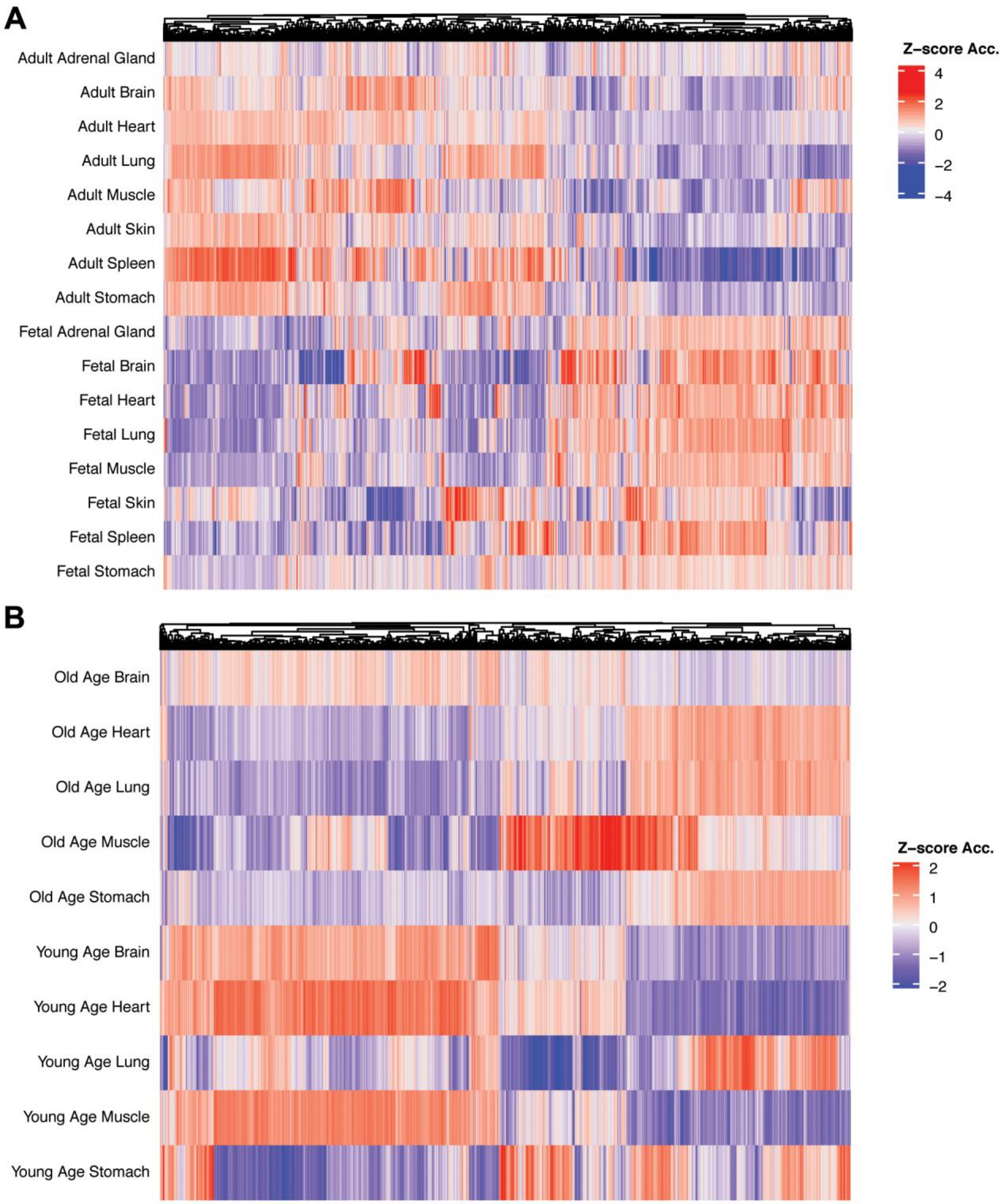

Supplementary Figure 6. Promoter accessibility heatmaps. Red-blue colour scale indicates increased/decreased accessibility, z-score normalized per-column. (**A**) Promoter accessibility differences between fetal and adult tissue samples, for significantly-altered promoters (adj. p-val < 0.05). (**B**) Promoter accessibility differences between young-age and old-age adult tissue samples, for significantly-altered promoters (adj. p-val < 0.05).

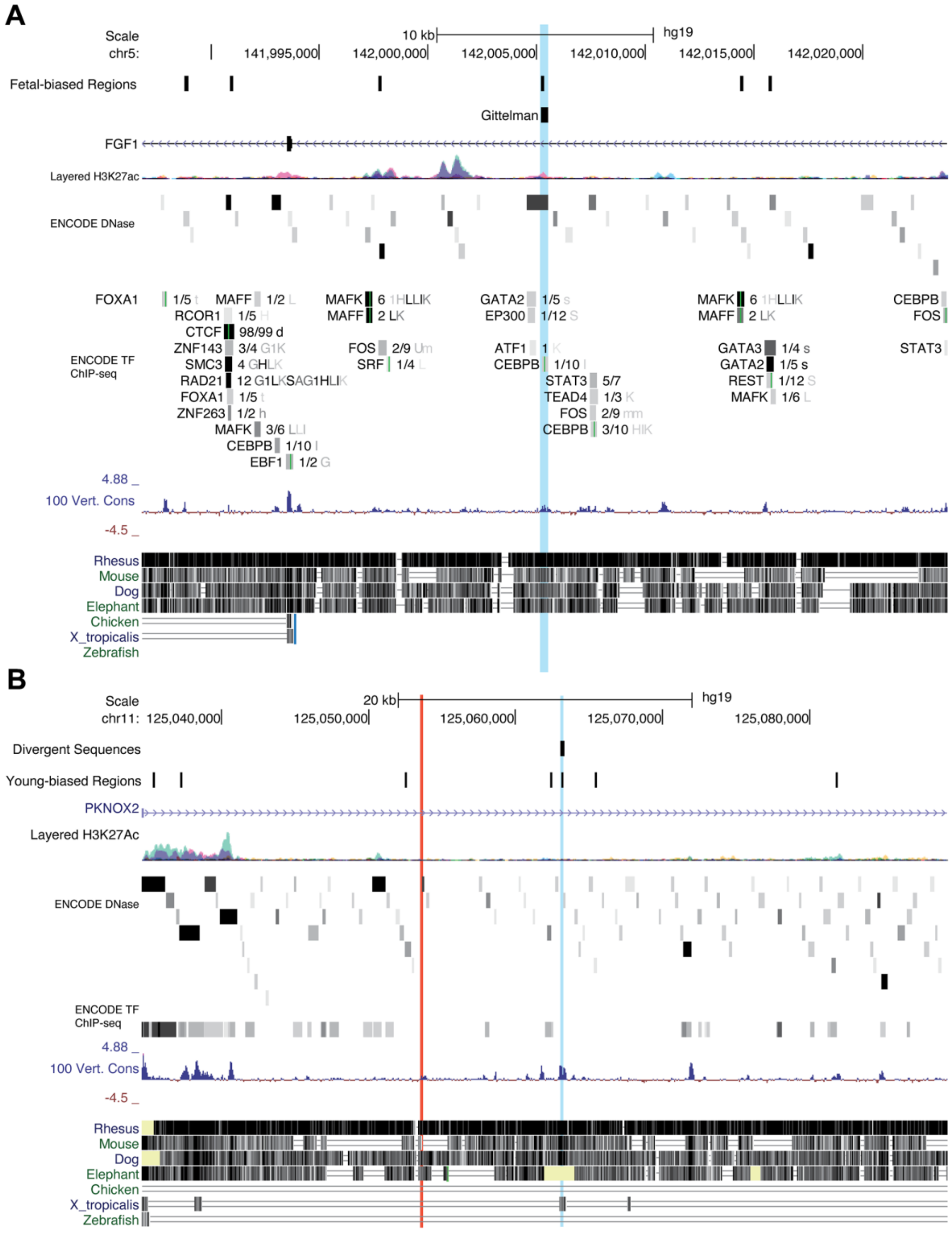

Supplementary Figure 7. UCSC genome screenshots for two representative human divergent-sequence loci. Additional tracks (top to bottom): Layered H3K27ac signal from ENCODE datasets, layered DNase-I hypersensitivity sites from ENCODE datasets, aggregated ENCODE transcription-factor ChIP-seq data, phyloP100ways conservation track (per-bp), multiple-sequence alignment to human reference sequence. (**A**) A human-accelerated region [20] (top track – highlighted in light blue) intersects a region losing accessibility in adult tissue (bottom track) intronic to the *FGF1 gene* (and which also has promoter-capture data to suggest promoter contact). Also intersects a possible CEBPB binding site (ENCODE TF-ChIP-seq track). (**B**) A human-accelerated region [20] (top track - highlighted in light blue) intersects a region losing accessibility in old-age adult tissue (bottom track) intronic to the *PKNOX2 gene* (and which also has promoter-capture data to suggest promoter contact). Upstream of this region lies the variant rs590211(highlighted in red), which has been associated with human-longevity via GWAS studies [25, 26].

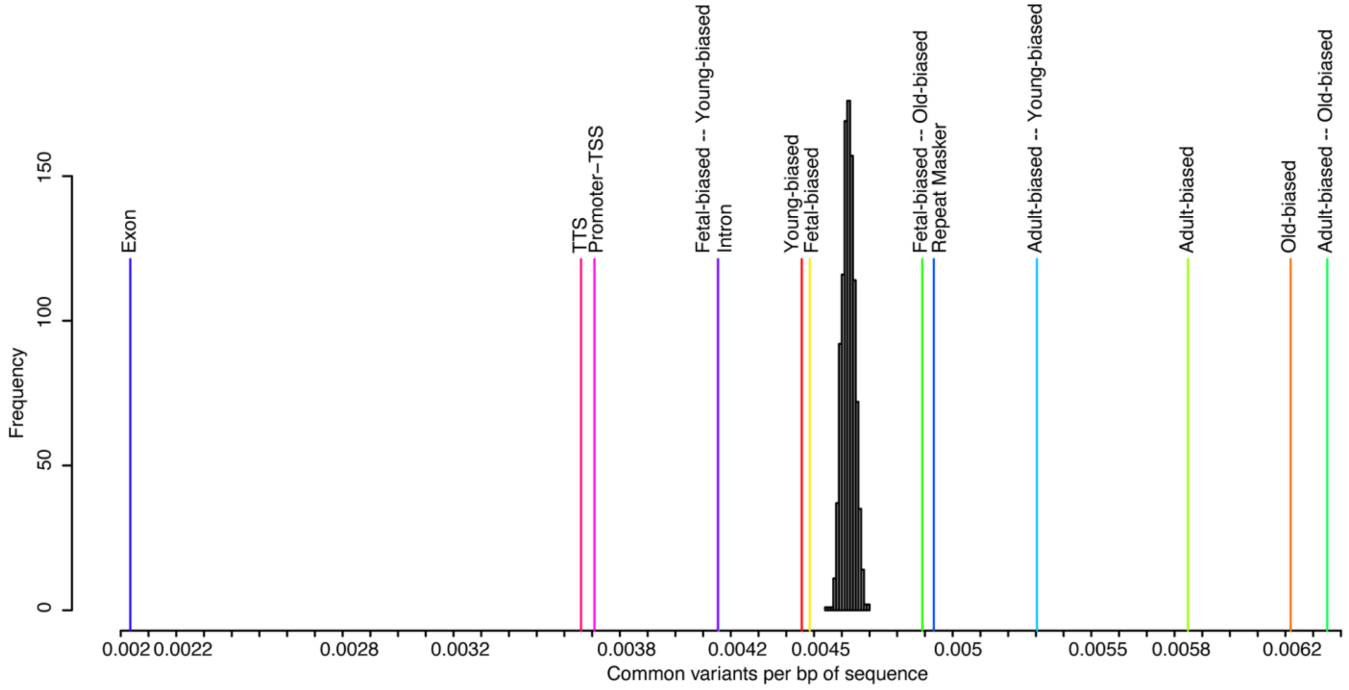

Supplementary Figure 8. Chimpanzee genomic distribution plot. Counts of chimpanzee common variants per bp of sequence for region sets were compared to random region sets along with other genomic features; labels correspond to results in Supplementary Table 3.
